# Supplementary material for: A genomic toolkit for winged bean Psophocarpus tetragonolobus
Source: Nat Commun. 2024 Mar 1;15:1901. doi: 10.1038/s41467-024-45048-x (PMC10907731; doi:10.1038/s41467-024-45048-x)
Supplement: Supplementary file 1 — Supplementary Information [file 41467_2024_45048_MOESM1_ESM.pdf]

A genomic toolkit for winged bean *Psophocarpus tetragonolobus*

Ho *et al.*

Supplementary Table 1. Types of transcription factors identified as a percentage of the total.

Type	%	Type	%
bHLH	9.32	ARR-B	0.79
MYB	10.36	ZF-HD	0.98
C2H2	5.85	HB-other	0.55
MYB_related	4.27	CAMTA	0.55
WRKY	5.55	DBB	0.55
ERF	6.28	NF-YA	0.55
NAC	5.18	SRS	0.61
bZIP	3.96	GRF	0.61
G2-like	3.05	NF-YC	0.43
GRAS	2.50	Nin-like	0.49
HD-ZIP	3.17	YABBY	0.55
LBD	2.74	BES1	0.43
FAR1	2.13	CPP	0.37
C3H	2.25	E2F/DP	0.43
Trihelix	2.38	BBR-BPC	0.24
B3	2.25	EIL	0.24
Dof	2.01	LSD	0.24
M-type_MADS	1.46	VOZ	0.18
MIKC_MADS	1.83	Whirly	0.18
TALE	1.77	GeBP	0.24
GATA	1.52	HB-PHD	0.18
HSF	1.58	NF-X1	0.06
ARF	1.52	NZZ/SPL	0.12
TCP	1.58	RAV	0.12
AP2	1.52	S1Fa-like	0.18
SBP	1.34	HRT-like	0.06
CO-like	0.85	LFY	0.06
NF-YB	0.85	STAT	0.06
WOX	0.85		

Supplementary Table 2. AMOVA analysis and PhiST (Φ) using Eucidean distances and ‘farthest neighbor’ algorithm with permutation test using Monte Carlo method.

By subpopulation

Variation	df	SS	MS	Sigma	%	Phi	*p*-value
Between pop	4	58341.0	14585.2	259.5	19.4	0.6863	>0.001
Between samples within pop	125	216714.9	1733.7	657.3	49.2	0.6106	>0.001
Within samples	130	54487.5	419.1	419.1	31.4	0.1943	<0.001
Total variation	259	329543.4	1272.4	1335.9	100.0		

By country of origin

Variation	df	SS	MS	Sigma	%	Phi	*p*-value
Between pop	17	69889.5	4111.1	174.3	13.4	0.6775	>0.001
Between samples within pop	112	205166.4	1831.8	706.4	54.3	0.6276	>0.001
Within samples	130	54487.5	32.2	419.1	32.2	0.1341	<0.001
Total variation	259	329543.4	100.0	1299.7	100.0		

Supplementary Table 3. Pairwise values of Nei's G_{ST} among Subpopulation 1 (Q1), Subpopulation 2 (Q2), Subpopulation 3 (Q3), Subpopulation (Q4).

	**Q2**	**Q3**	**Q4**
**Q1**	0.2163	0.4561	0.2968
**Q2**		0.3393	0.1596
**Q3**			0.3810

Supplementary Table 4. Number of SNP per pseudo-molecules after filtering used in LD decay analysis (*p*-value determined by two-sided Fisher's Exact test).

Chromosome	No. of SNP	number of SNP pair in LD at $p<0.05$
Chr01	1751	18648
Chr02	1349	14700
Chr03	1455	13199
Chr04	1249	12461
Chr05	1405	15351
Chr06	1104	11753
Chr07	849	8007
Chr08	769	8671
Chr09	603	5221
SCF	33	

Supplementary Table 5. Primers used for the gene expression studies.

Primer name	Sequence (5' -> 3')
PtMYB113a_F2	GACAATGCAGGAAACATTGGC
PtMYB113a_R2	TCTCCTAGCTAGGTGAGAAGAAAATC
PtMYB113b_F	CAAGAGAAACTCGTTAGTAGTTCC
PtMYB113b_R	ATTGAAATAAGGTTTTGATCCGAAACT
PtELFa_F	AGCTGCTGAGATGAACAAGAGG
PtELFa_R	TCTCAAACTTCCACAAGGCAATATC
PtTUB6_F	GAGTGCATGGTCCTTGATAATGA
PtTUB6_R	CTCATAGTAGCAGAGATCAAGTGG

a.

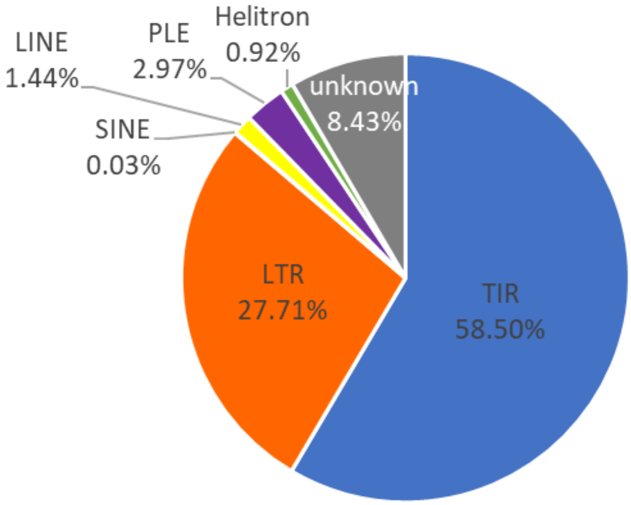

.

b.

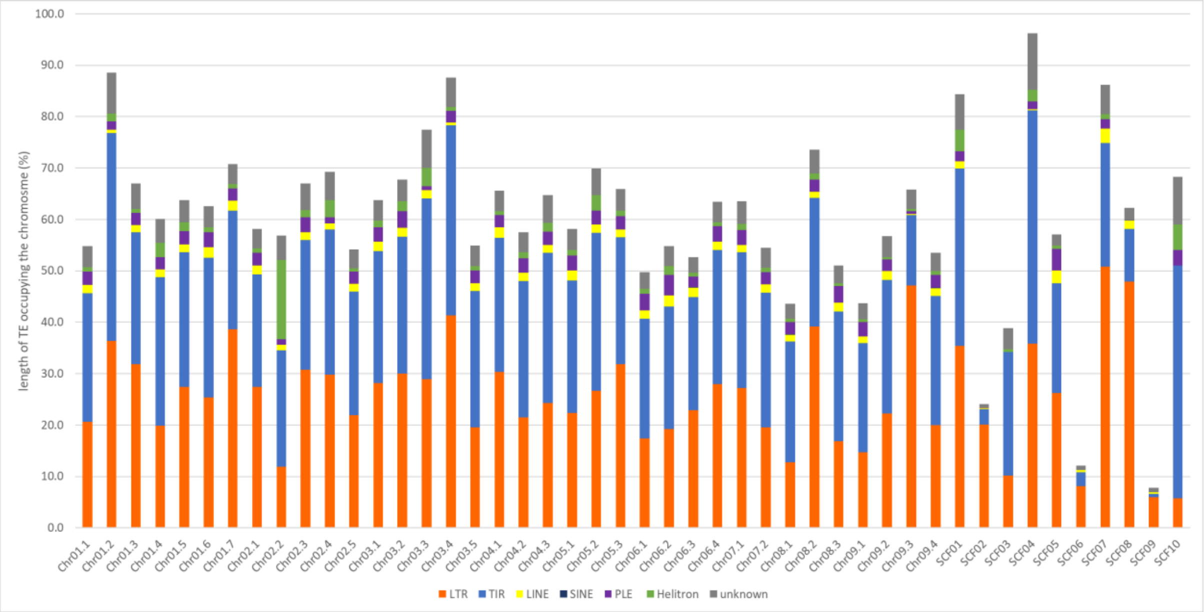

Supplementary Figure 1. Summary of the characterisation of repetitive elements. (a) Types of repetitive elements identified in winged bean genome and (b) their length distributions (in %) across the genome.

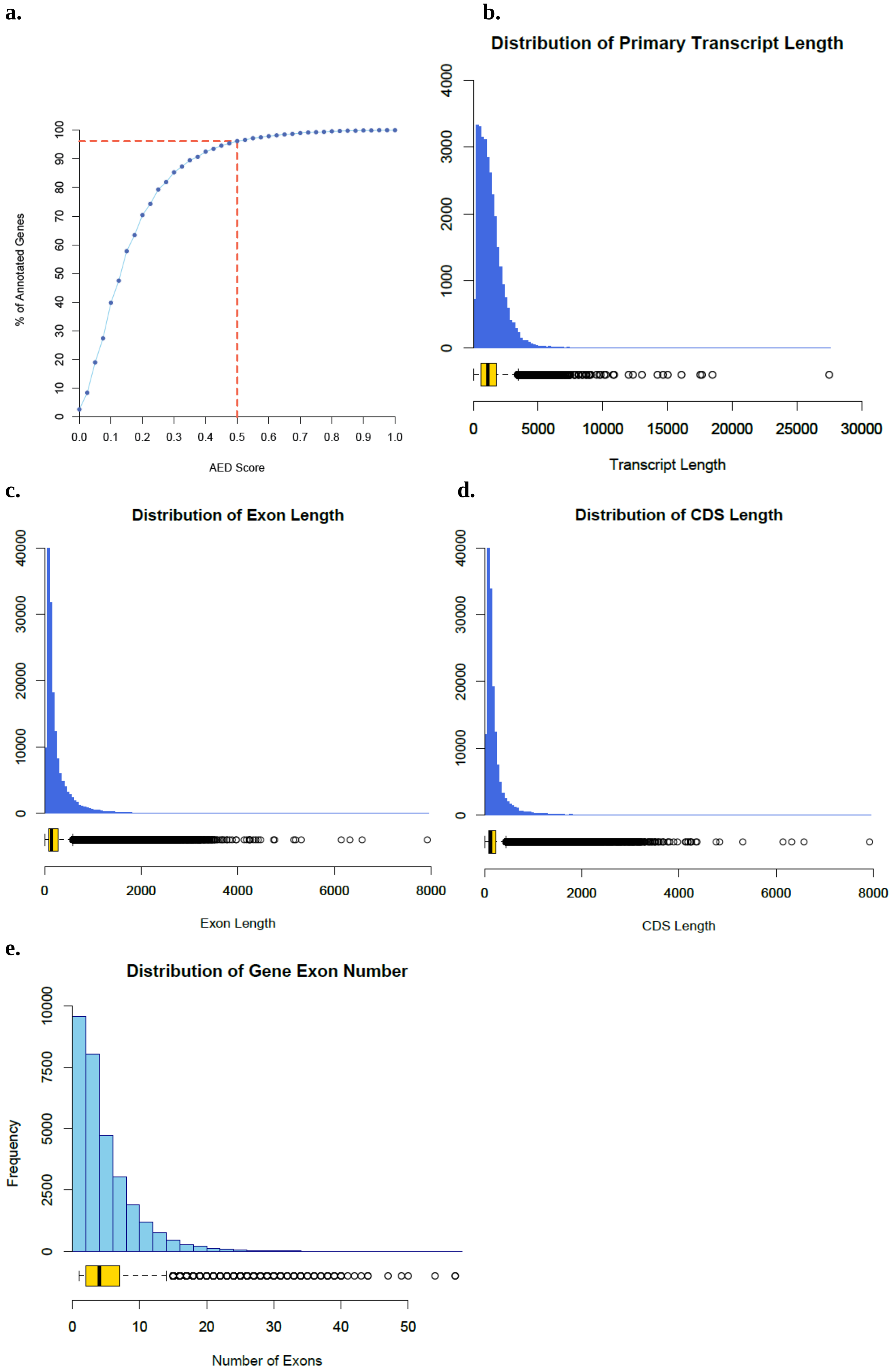
a.
% of Annotated Genes
AED Score
b.
Distribution of Primary Transcript Length
Transcript Length
c.
Distribution of Exon Length
Exon Length
d.
Distribution of CDS Length
CDS Length
e.
Distribution of Gene Exon Number
Frequency
Number of Exons

Supplementary Figure 2. Characterisation of gene models in the winged bean genome. (a) Annotation Edit Distance (AED) score as a measure of annotation quality in terms of specificity and sensitivity. High quality annotations are those of 0.3 and below[1]. An overview of (b) primary transcript lengths, (c) exon length, (d) CDS length in bp and the frequencies of the annotated gene models, (e) distribution of exon number per gene.

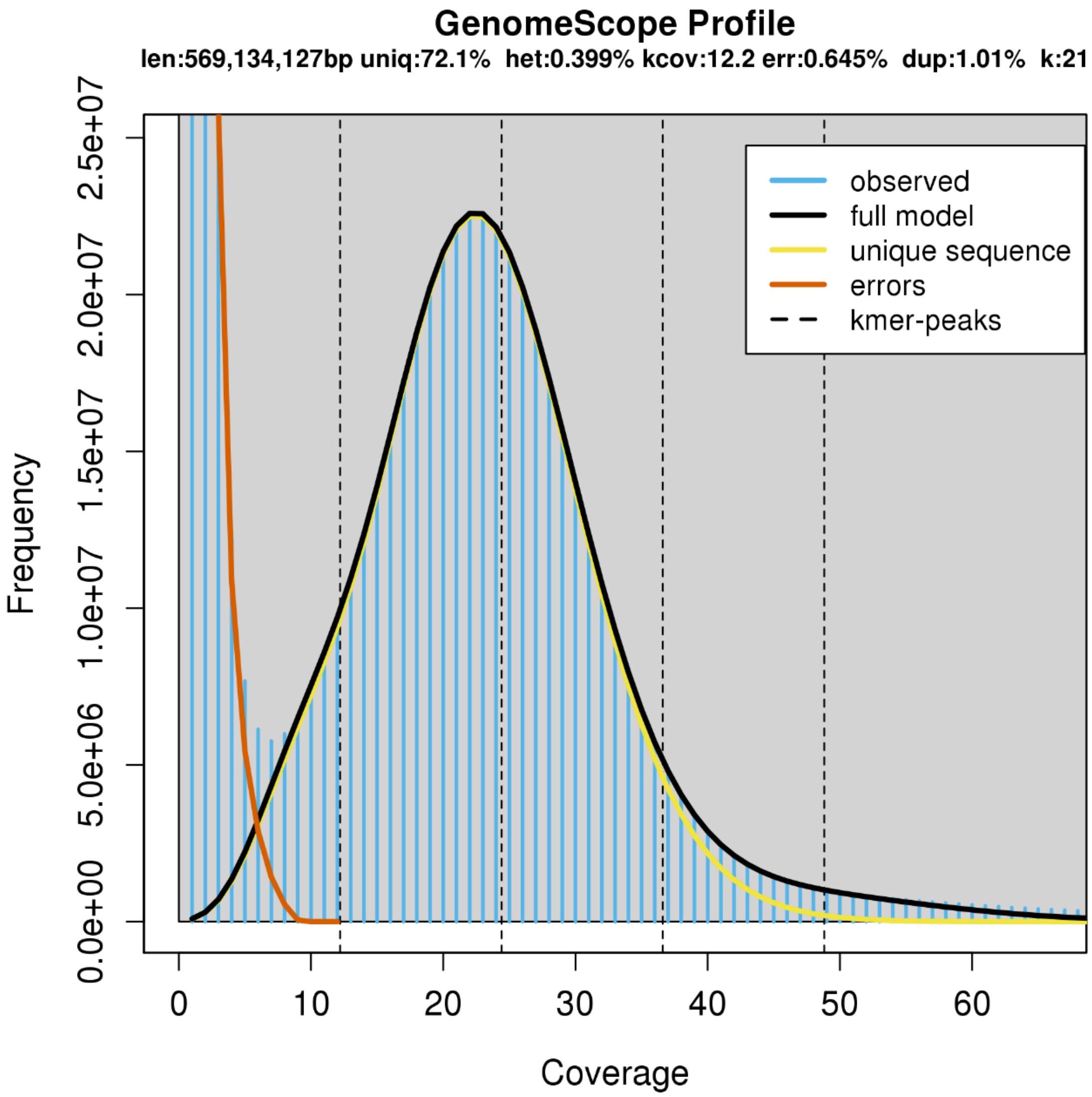

Supplementary Figure 3. *K*-mer analysis. The K-mer analysis has suggested a genome size of 569 Mb for winged bean.

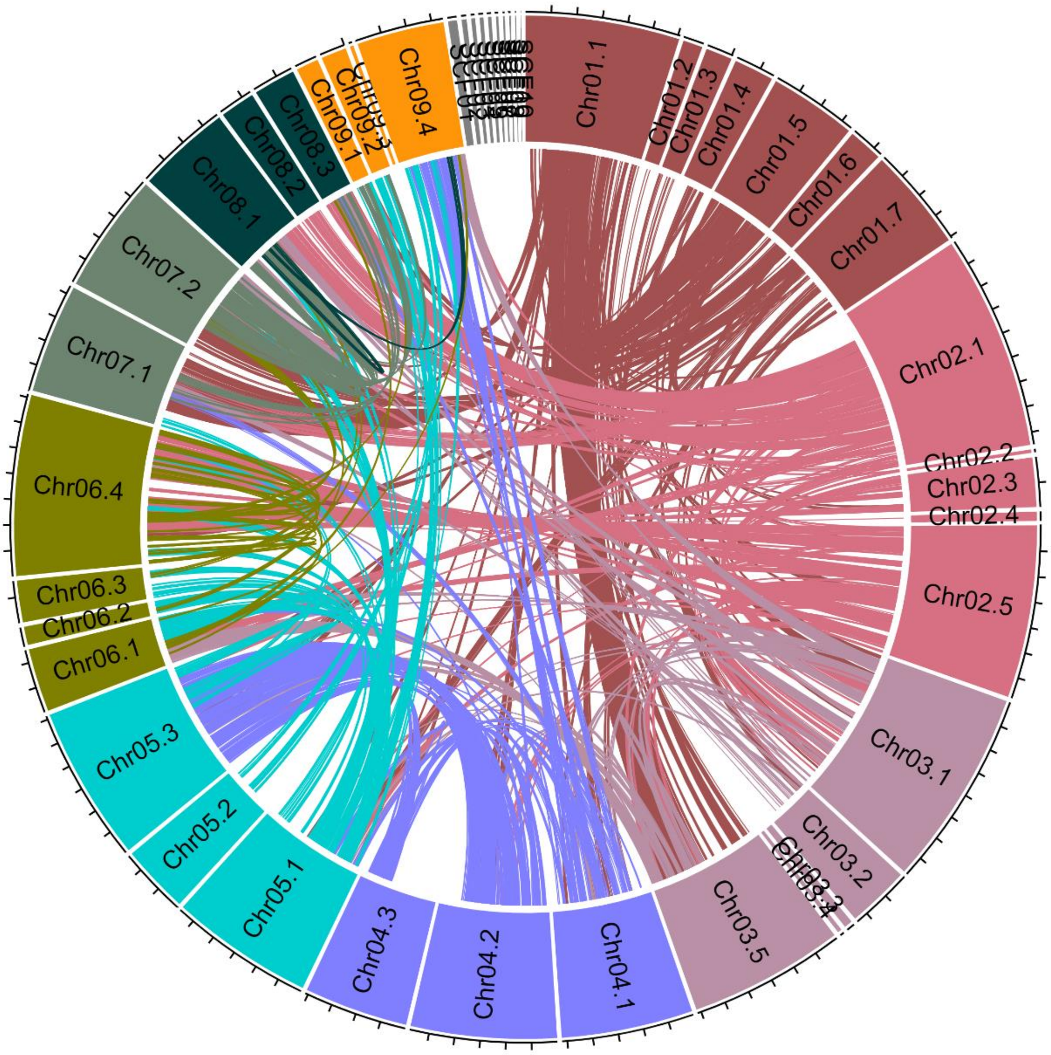

Supplementary Figure 4. The syntenic blocks in winged bean. The paralogues identified in blocks among the winged bean chromosomes.

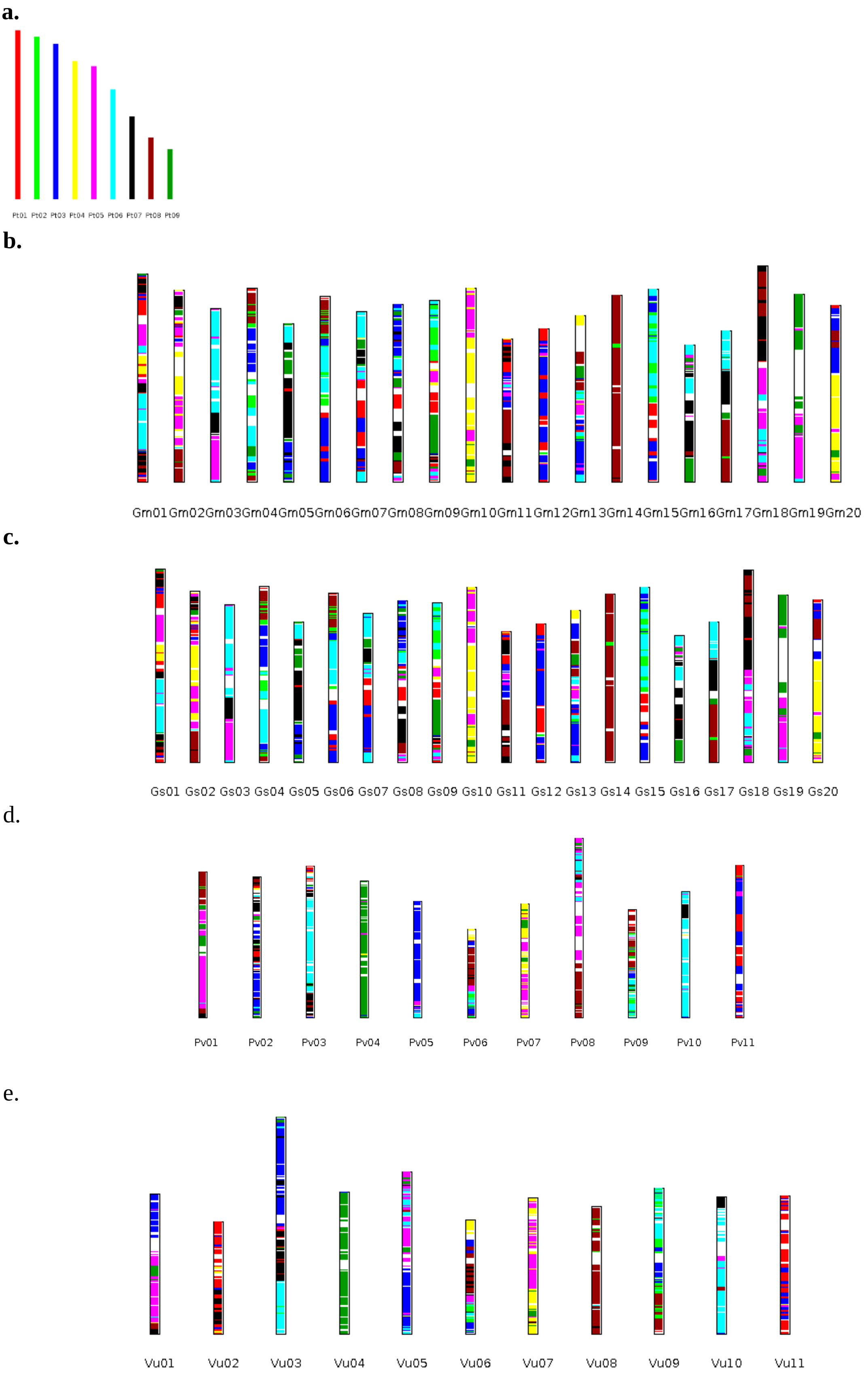

Supplementary Figure 5. Syntenic relationship of winged bean with closely related legume species. Chromosome rearrangement observed between (a) winged bean and closely related species, (b) *G. max*, (c) *G. soja*, (d) *P. vulgaris*, (e) *V. unguiculata*.

a.

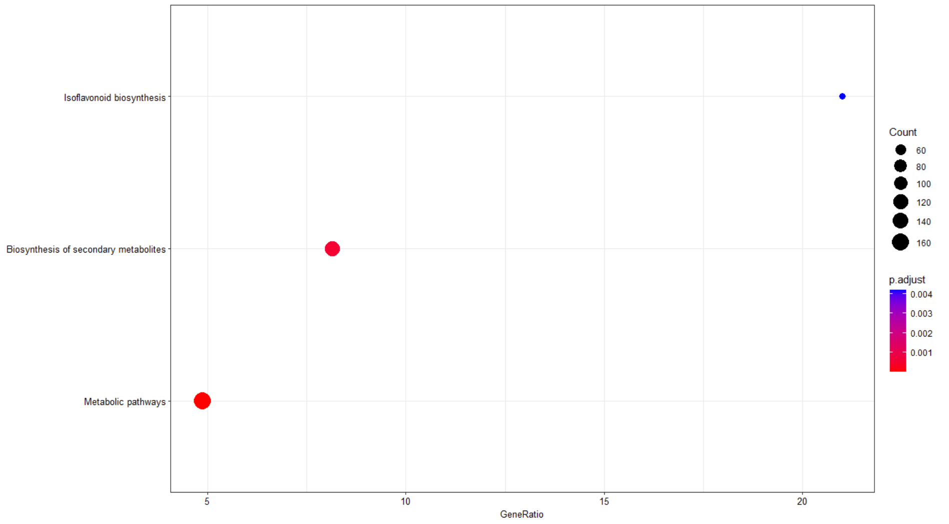

b.

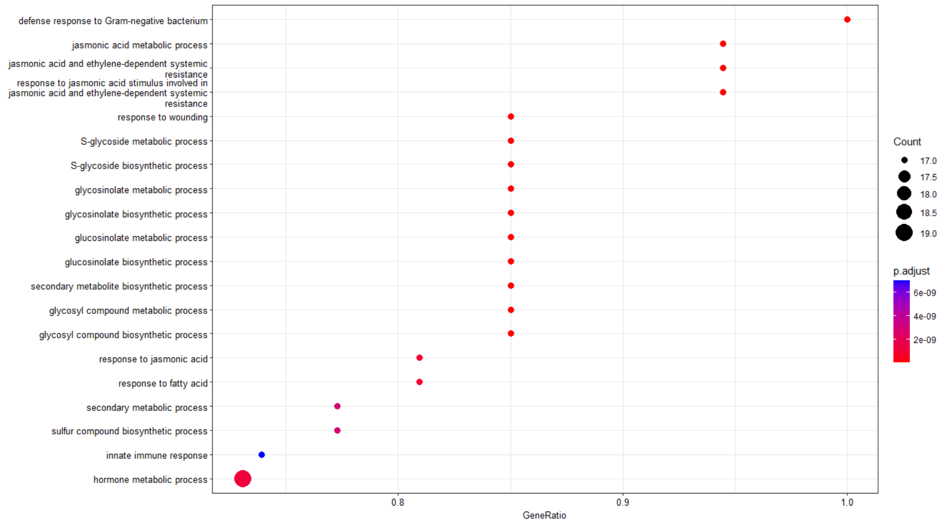

c.

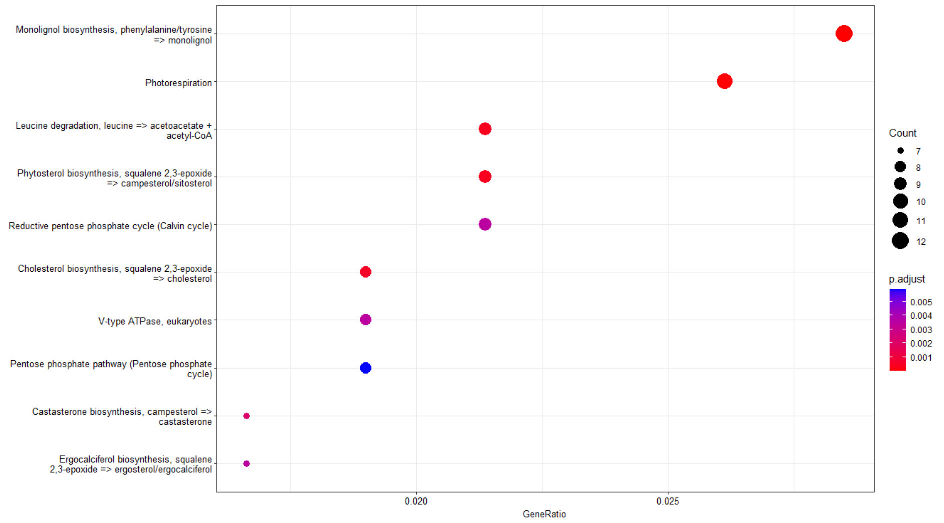

Supplementary Figure 6. Enrichment analysis of the expanded gene family and duplicated genes. Enrichment analysis of the (a-b) expanded gene families ($p < 0.05$, p adjusted method = Benjamini-Hochberg) and (c) duplicated genes ($p < 0.01$, p adjusted method = Benjamini-Hochberg).

a.

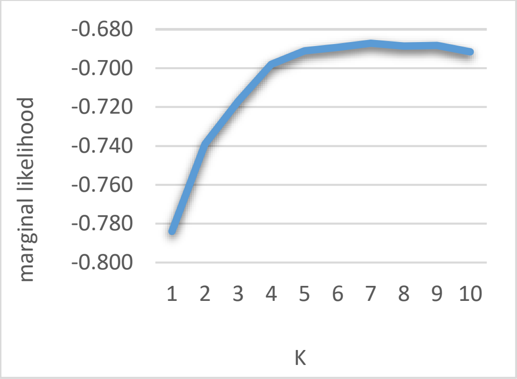

-0.680
-0.700
-0.720
-0.740
-0.760
-0.780
-0.800
marginal likelihood
1 2 3 4 5 6 7 8 9 10
K

b.

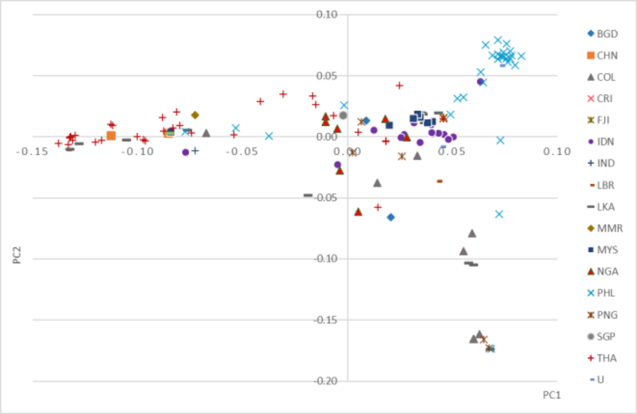

0.10
0.05
0.00
-0.05
-0.10
-0.15
-0.20
-0.15 -0.10 -0.05 0.00 0.05 0.10
PC1
PC2
BGD
CHN
COL
CRI
FJI
IDN
IND
LBR
LKA
MMR
MYS
NGA
PHL
PNG
SGP
THA
U

c.

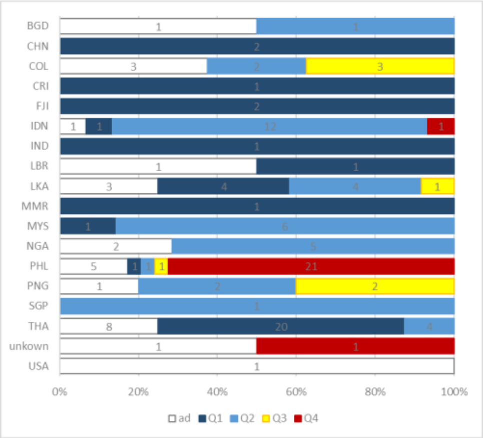

BGD
CHN
COL
CRI
FJI
IDN
IND
LBR
LKA
MMR
MYS
NGA
PHL
PNG
SGP
THA
unkown
USA
0% 20% 40% 60% 80% 100%
ad Q1 Q2 Q3 Q4

d.

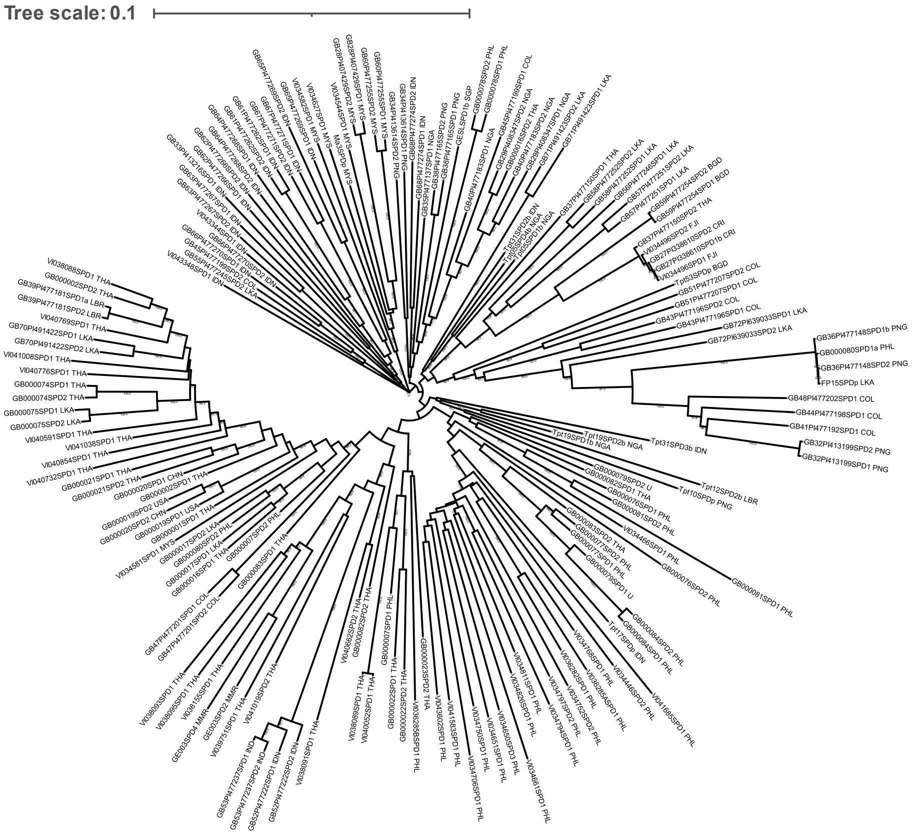

Tree scale: 0.1

Supplementary Figure 7. Genetic diversity analysis. (a) The marginal likelihood when *K* ranged from one to ten. (b) PCoA analysis and (c) bar chart of the germplasm coloured by their origin of collection demonstrates the clustering does not correspond very well with country of origin. BGD: Bangladesh; CHN: China; COL: Colombia, CRI: Costa Rica; FJI: Fiji; IDN: Indonesia; IND: India; LBR: Liberia; LKA: Sri Lanka; MMR: Myanmar; MYS: Malaysia; NGA: Nigeria; PHL: Philippines, PNG: Papua New Guinea; SGP: Singapore; THA: Thailand; U: unknown. (d) NJ tree demonstrating clustering of all germplasms listed in Supplementary Data 7 including biological replicates using DArTseq SNPs.

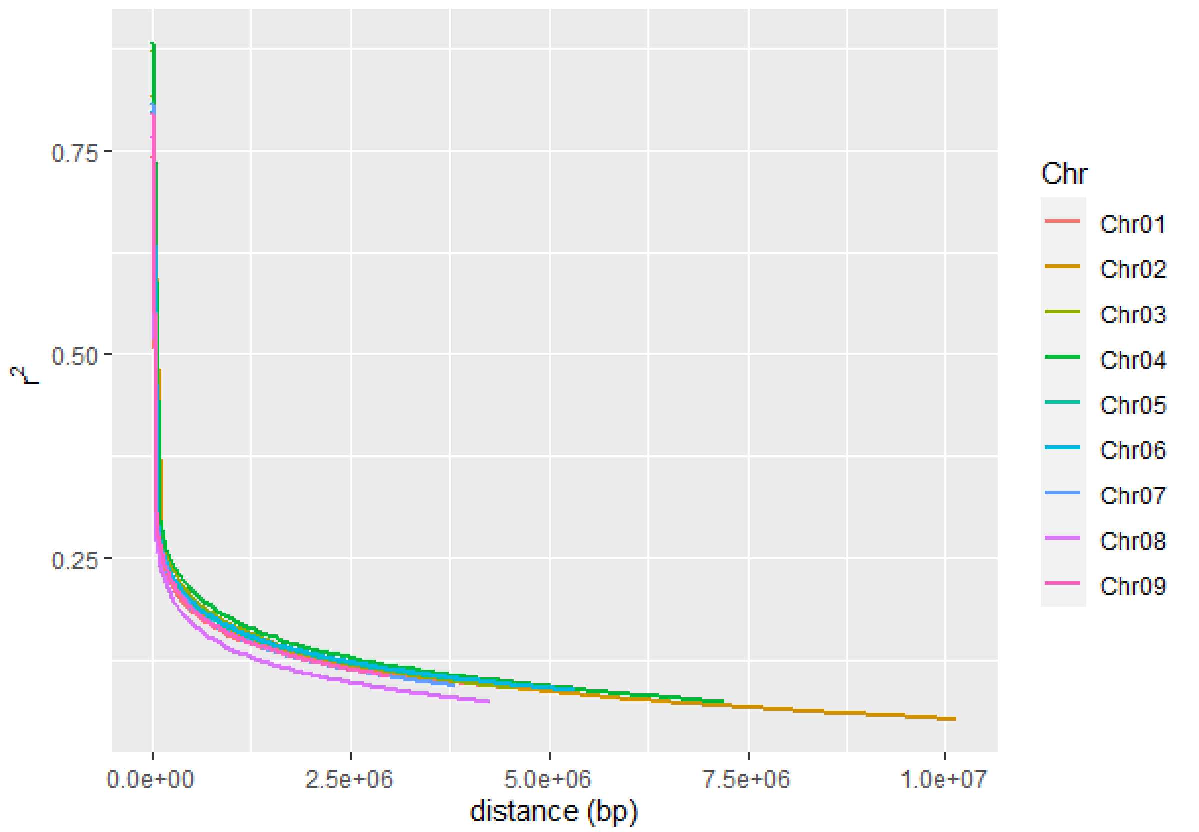

Supplementary Figure 8. Linkage disequilibrium analysis. LD decay plot, according to pseudochromosome.

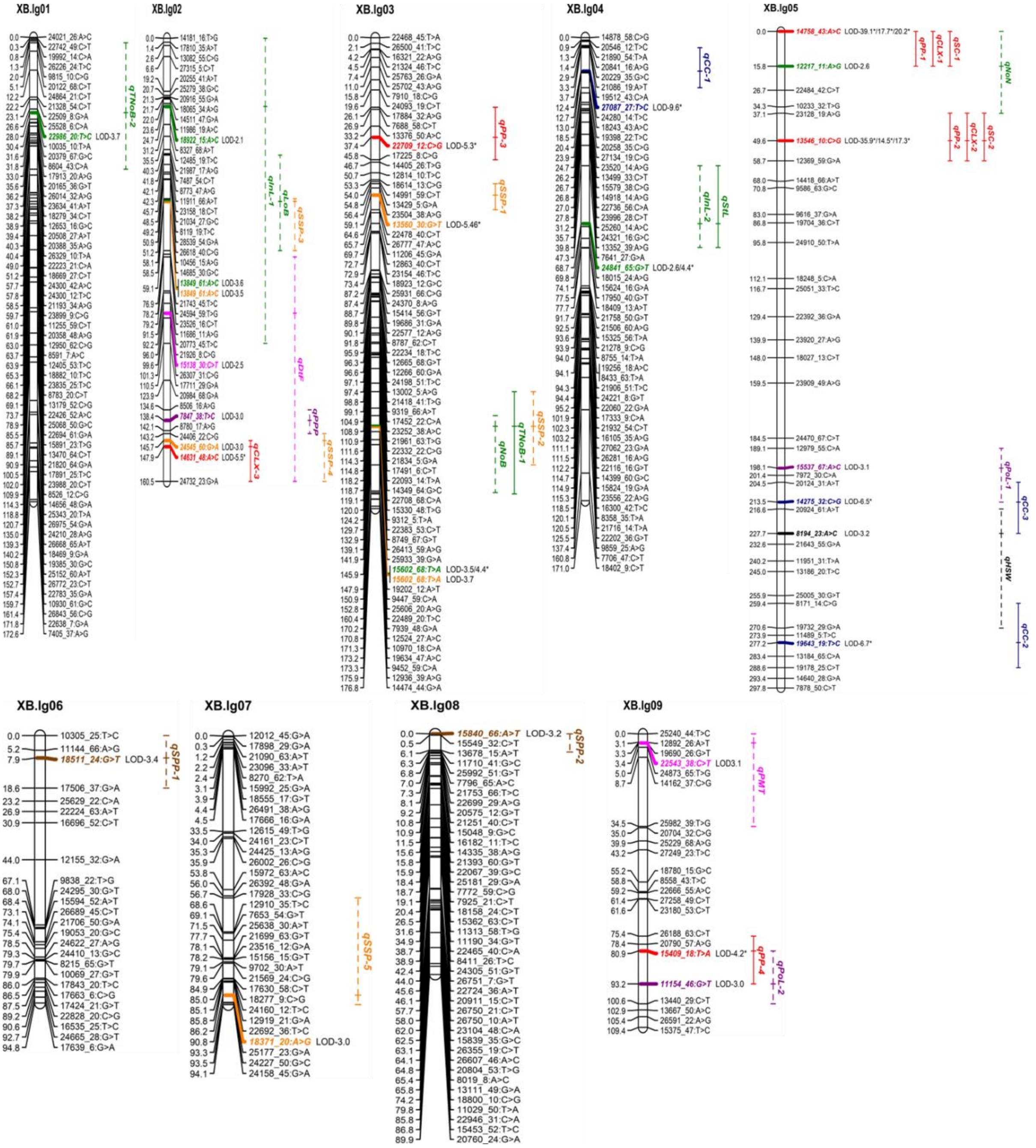

Supplementary Figure 9. **Linkage groups and QTL of XB population.** TNoB: total branch number per plant; InL: internode length; LoB: length of branch; DtF: days to first open flower; PPP: mature pods per plant; CLX: calyx colour; PP: specks presence/ pod pigmentation; NoB: branch number per plant; StL: stem length; SC: seed coat colour; NoN: number of nodes; PoL: dry pod length; HSW: 100-seed weight; SPP: seeds per pod; PMT: pod maturing time; SSP: seed storage protein percentage; CC: chlorophyll content. Vertical lines represent the 2-LOD support interval for significant QTL (LOD ≥ GW-threshold). Dashed lines represent QTL support intervals for putative QTL.

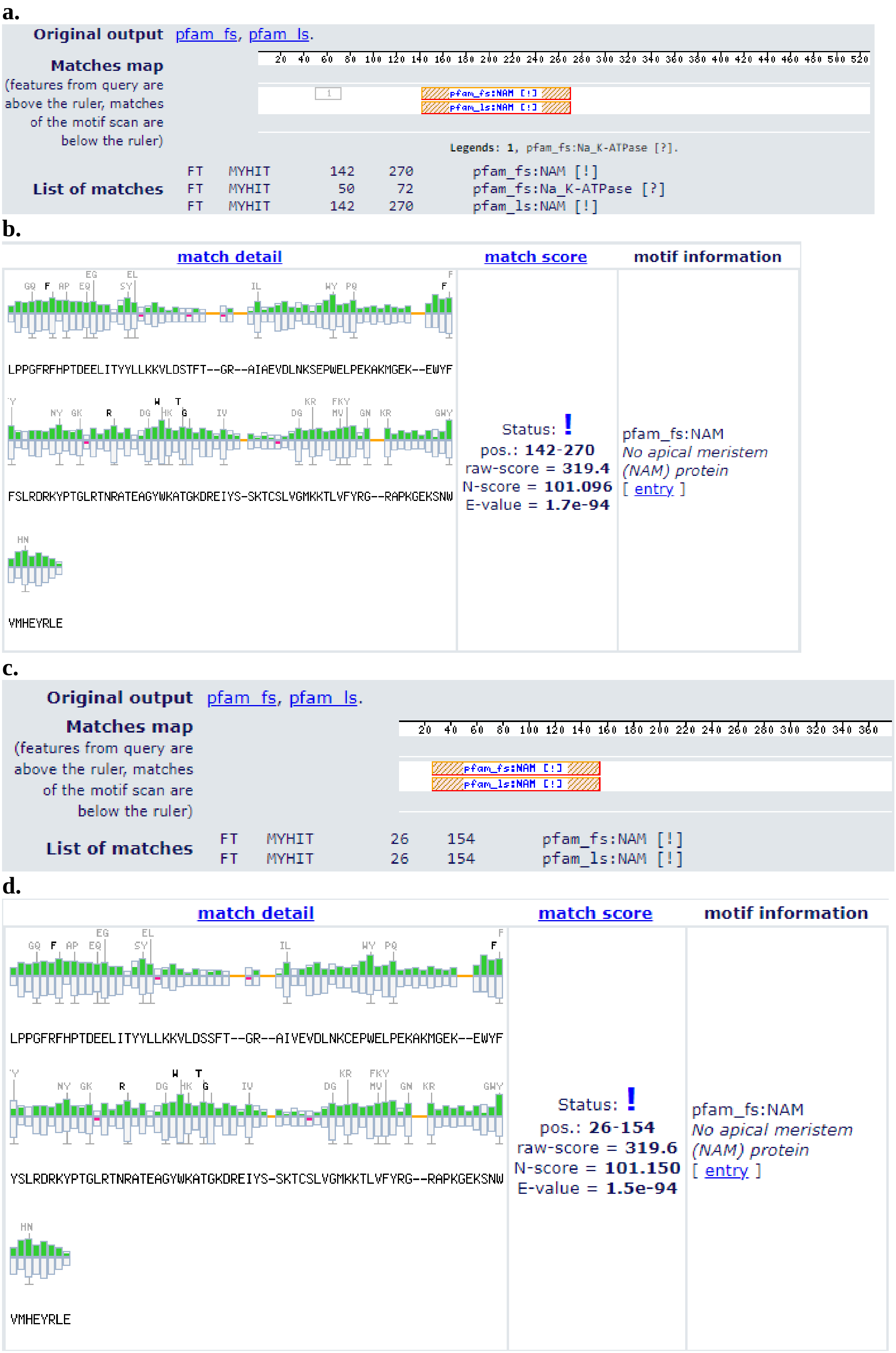

Supplementary Figure 10. Motif scan analysis for plant architecture related gene. The motif scan result of (a, b) Psote03G0327500.1 and (c,d) Psote01G0148300.1 using Motif Scan of MyHits (https://myhits.sib.swiss/cgi-bin/PFSCAN), in terms of the genomic position and the amino acid conservation.

a.

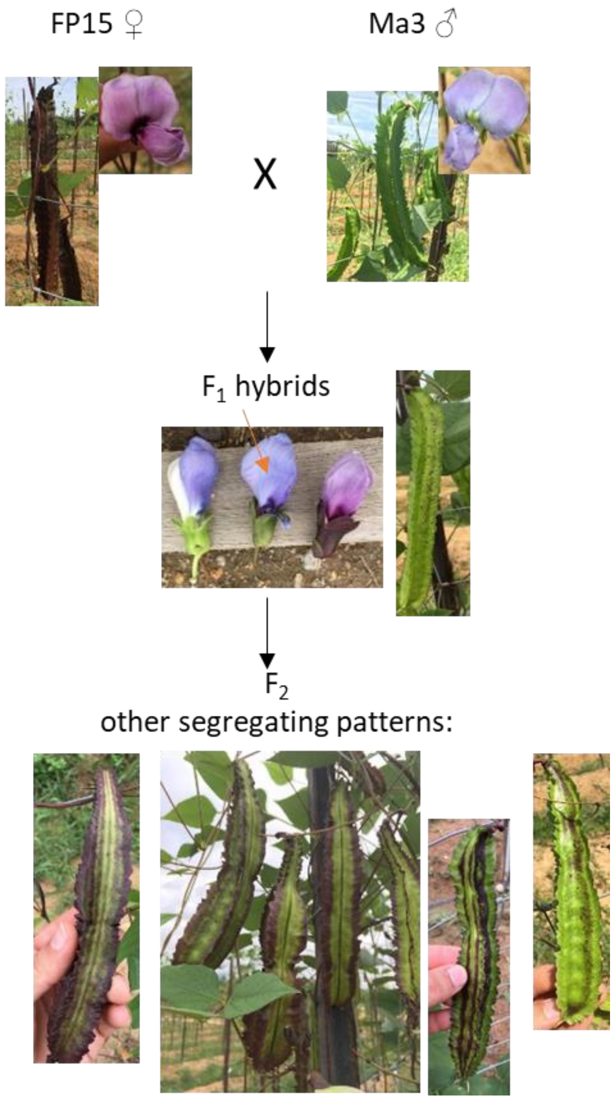

b.

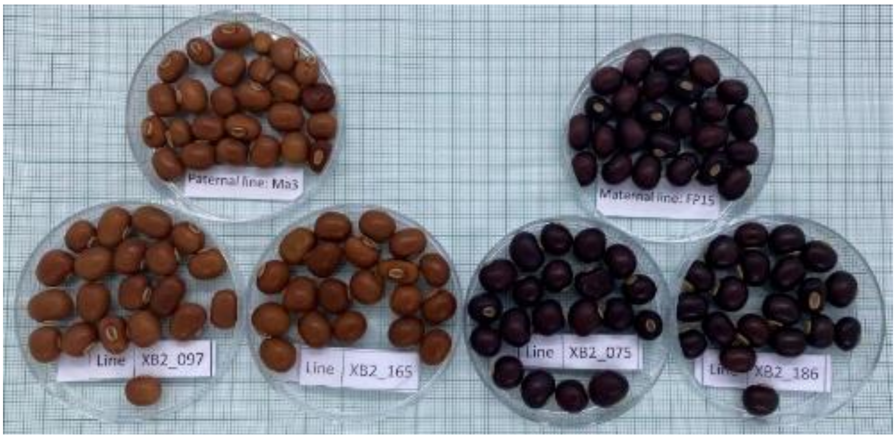

Supplementary Figure 11. Pigment inheritance observed in the XB population. (a) While the petal colour of both genotypes is not easily distinguishable (the Ma3 genotype has light violet flowers whilst the FP15 genotype bore fuchsia-coloured flowers), their sepal colour does contrast. A green calyx was observed in the Ma3 genotype whilst dark purple was seen in FP15. (b) Pigmentation variation in the mature seed coat.

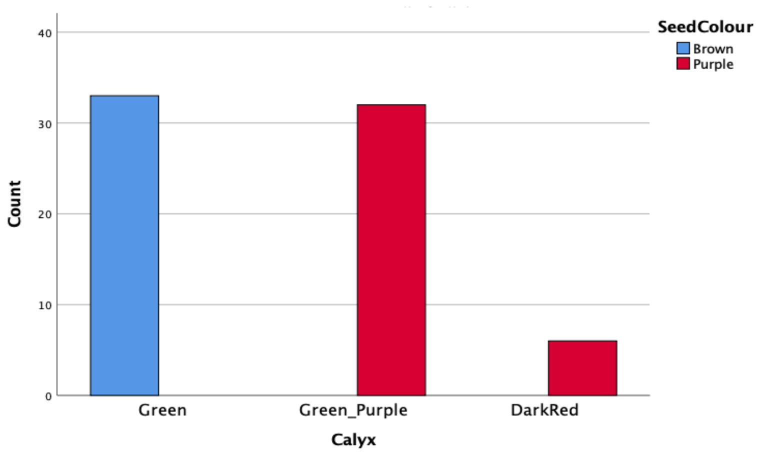

Supplementary Figure 12. Relationship of seed coat colour vs calyx colour. Seed coat colour observed in the F_2 of XB population in relations to calyx colour.

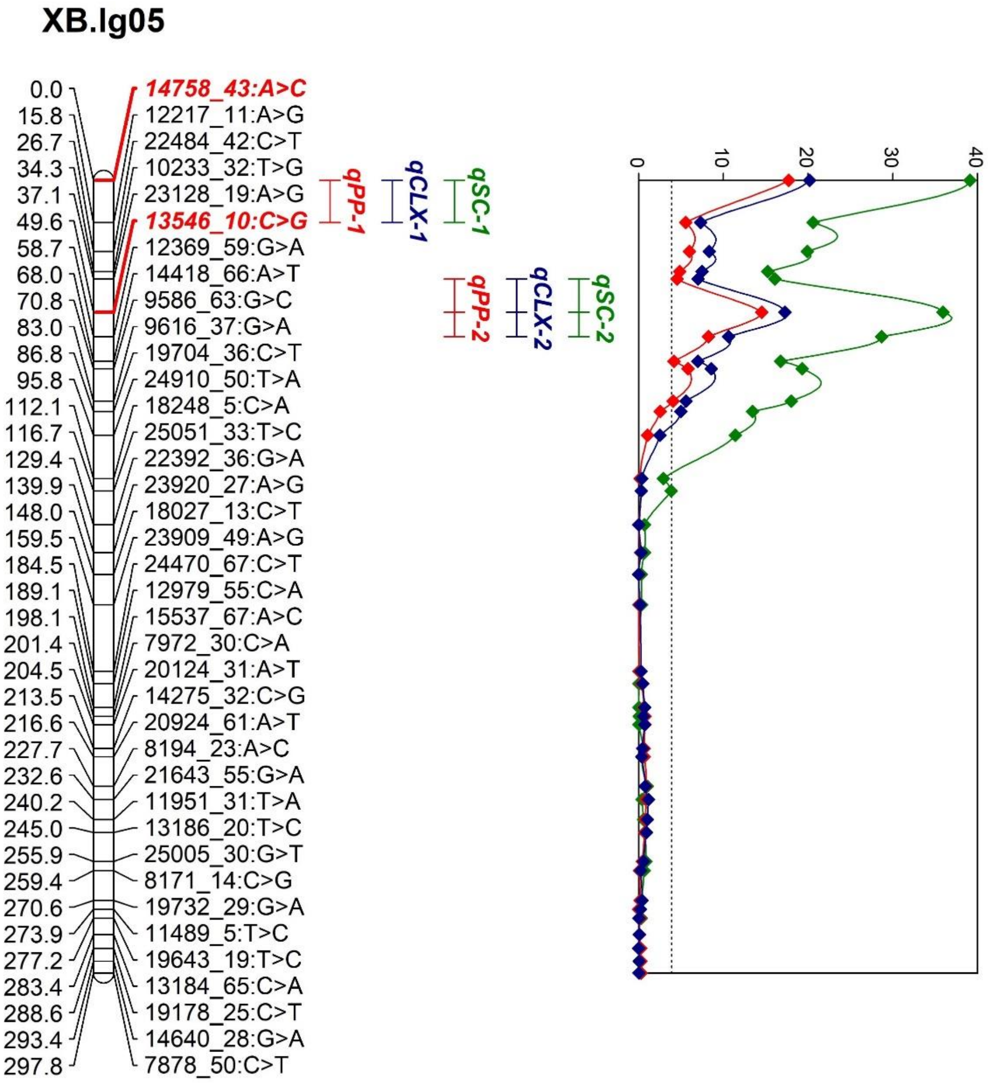

Supplementary Figure 13. Pigmentation loci. The LOD score of seed coat colour (*qSC*), pod pattern (*qPP*) and calyx colour (*qCLX*) on Pt05 (Chr05.1)/XB.lg05. The major peaks were designated as *P1* and *P2* alleles.

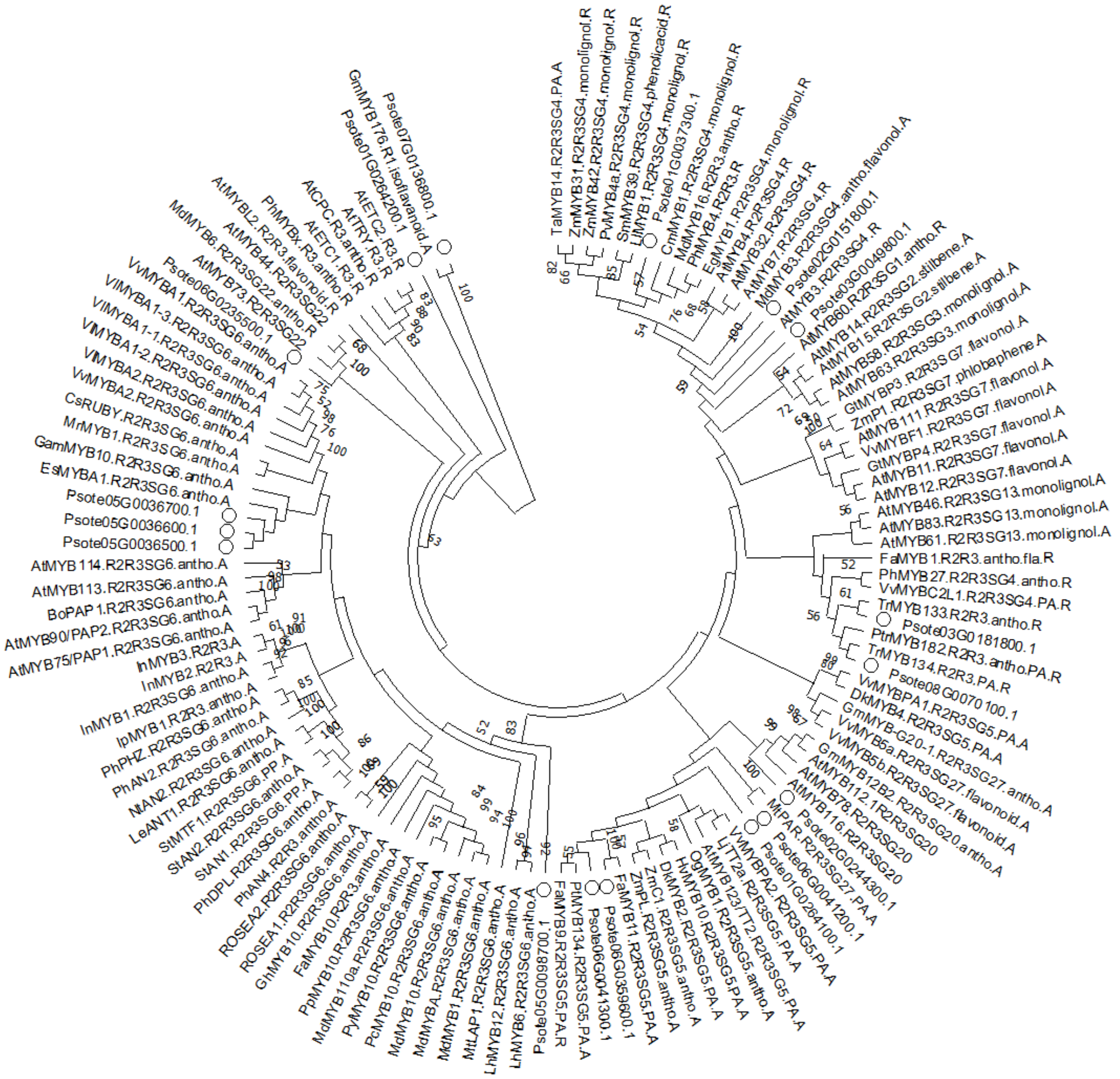

Supplementary Figure 14. Characterisation of MYB genes involved in the flavonoid biosynthetic pathway. Characterisation of 17 winged bean MYB genes (marked with circles) with the designated MYB subgroups as previously reported[2-4] using NJ method (bootstrap values with 5000 replicates are shown as percentage next to the branches), SG: subgroup, antho: anthocyanin, PA: proanthocyanidin, A: activator, R: repressor. The species and accession numbers of labelled MYB proteins are listed in Supplementary Data 16.

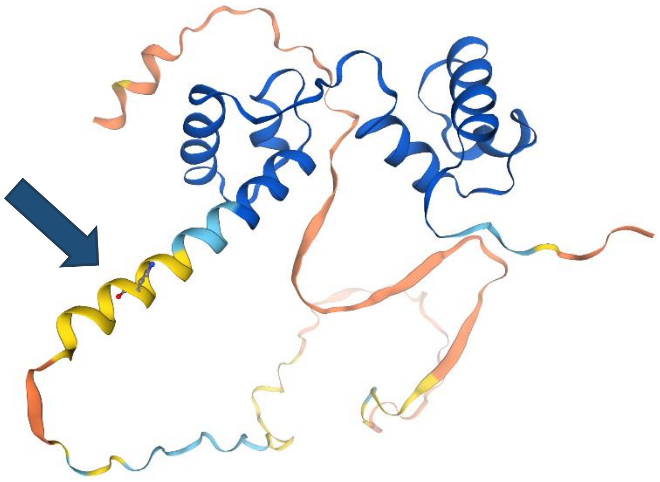

Supplementary Figure 15. The 3D structure of PtMYB113b with the three amino acid insertion/deletion indicated by the arrow.

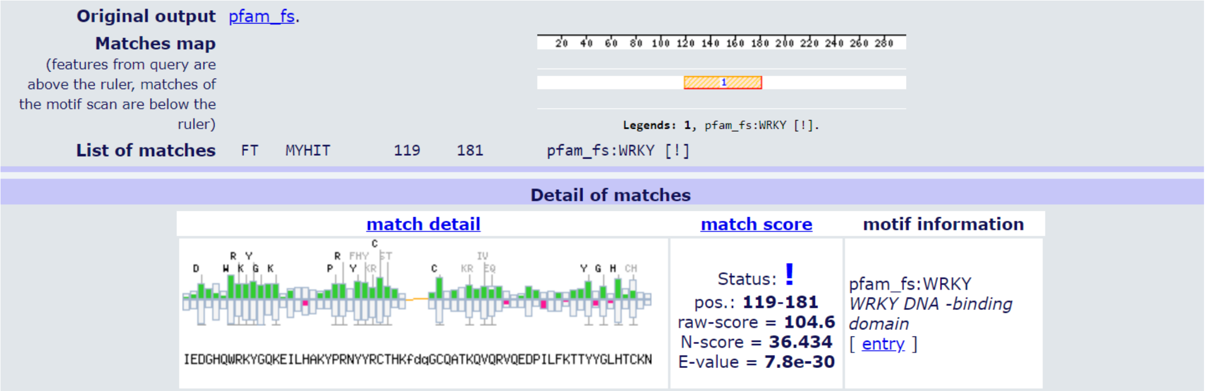

Supplementary Figure 16. The position of WRKY domain in PtWRKY70.

Supplementary references

1. Hunt, S. P. *et al.* A chromosome-scale assembly of the garden orach (*Atriplex hortensis* L.) genome using Oxford Nanopore sequencing. *Front. Plant Sci.* **11**, (2020).
2. Stracke, R., Werber, M. & Weisshaar, B. The R2R3-MYB gene family in *Arabidopsis thaliana*. *Curr. Opin. Plant Biol.* **4**, 447–456 (2001).
3. Deng, J. *et al.* Multiple MYB activators and repressors collaboratively regulate the juvenile red fading in leaves of sweetpotato. *Front. Plant Sci.* **11**, 941 (2020).
4. Liu, J., Osbourn, A. & Ma, P. MYB transcription factors as regulators of phenylpropanoid metabolism in plants. *Mol. Plant* **8**, 689–708 (2015).
