# Supplementary material for: A genomic toolkit for winged bean Psophocarpus tetragonolobus
Source: Nat Commun. 2024 Mar 1;15:1901. doi: 10.1038/s41467-024-45048-x (PMC10907731; doi:10.1038/s41467-024-45048-x)
Supplement: Supplementary file 3 — Description of Additional Supplementary Files [file 41467_2024_45048_MOESM3_ESM.pdf]

File Name: Supplementary Data 1
Description: Summary of the genome assembly statistics of ONT reads, Illumina reads and Bionano molecules generated.

File Name: Supplementary Data 2
Description: Summary of scaffolds assembled into nine pseudochromosomes from Genetic Map XT and XB.

File Name: Supplementary Data 3
Description: Summary of the types of identified transposable element across genome.

File Name: Supplementary Data 4
Description: Summary of the distribution of types of transposable element [in length (bp)] across genome.

File Name: Supplementary Data 5
Description: Summary of the identified duplicated genes and duplication types.

File Name: Supplementary Data 6
Description: Summary of orthogroups in legume species with *Vitis* being the outgroup species.

File Name: Supplementary Data 7
Description: The list of materials included in this study. Replicates as described in Materials and Methods were only analysed once.

File Name: Supplementary Data 8
Description: Statistics summary of genotypic richness, diversity and evenness in four subpopulations (Q1, Q2, Q3 and Q4).

File Name: Supplementary Data 9
Description: Summary of QTL results measured in XB populations.

File Name: Supplementary Data 10
Description: List of the identified albumin and globulin orthologues in winged bean.

File Name: Supplementary Data 11
Description: Summary of the expression levels (FPKM) of the identified seed storage protein genes and selected candidate genes in the pod tissues at 15 days, 30 days, 37 days and 45 days after anthesis.

File Name: Supplementary Data 12
Description: List of the candidate genes of qSSP-1 and their expression levels in four stages of developing pod tissues.

File Name: Supplementary Data 13
Description: The genome position of the candidate genes involved in architectural traits and their orthologues.

File Name: Supplementary Data 14
Description: List of genes (genomic location and expression level) involved in the anthocyanin biosynthesis pathway in four stages of developing pod tissues.

File Name: Supplementary Data 15
Description: List of protein sequences identified from the MYB domain search.

File Name: Supplementary Data 16
Description: List of MYB transcription factors used in the phylogenetic analysis.

File Name: Supplementary Data 17
Description: Amino acid alignment of R2R3 MYB transcription factor Subgroup 6 orthologues.

File Name: Supplementary Data 18
Description: Summary of the variants identified in the candidate genes (genome position and amino acid changes) among the parental lines.

File Name: Supplementary Data 19
Description: Summary of RNA sequencing libraries statistics.
