# Supplementary material for: A genomic toolkit for winged bean Psophocarpus tetragonolobus
Source: Nat Commun. 2024 Mar 1;15:1901. doi: 10.1038/s41467-024-45048-x (PMC10907731; doi:10.1038/s41467-024-45048-x)
Supplement: Supplementary file 20 — Supplementary Data 17 [file 41467_2024_45048_MOESM20_ESM.docx]

PpMYB10.R2R3SG6.antho.A ---------------------------------------------------MEGYD--LS 7

MdMYB1.R2R3SG6.antho.A ---------------------------------------------------MEGYNENLS 9

MdMYB10.R2R3SG6.antho.A ---------------------------------------------------MEGYNENLS 9

PcMYB10.R2R3SG6.antho.A ---------------------------------------------------MEGYNVNLS 9

PyMYB10.R2R3SG6.antho.A ---------------------------------------------------MEGYNVNLS 9

**Psote05G0036700.1 ------------------------------------------------SKSHMERSSISS 12**

**Psote05G0036500.1 ------------------------------------------------------MKESFG 6**

**Psote05G0036600.1 ------------------------------------------------------MEGSSG 6**

**Psote05G0036600.1_FP15 ------------------------------------------------------MEGSSG 6**

Glyma.18G261700 ------------------------------------------------------MEGPSG 6

Glyma.09G235300 ------------------------------------------------------MEGPSS 6

Glyma.18G262000 ------------------------------------------------------MEGSSG 6

Glyma.09G234900 ------------------------------------------------------MEGSSG 6

Glyma.09G235100 ------------------------------------------------------MEGSSG 6

StMTF1.R2R3SG6.PP.A --------------------------------------------------MNSTSMSSLG 10

LeANT1.R2R3SG6.antho.A --------------------------------------------------MNSTSMSSLG 10

Medtr5g079290 --------------------------------------------MSMDNKGKLNMEMTRG 16

Medtr5g078950 ------------------------------------------------------MDNTRA 6

Medtr5g078910 ------------------------------------------------------MEKTRS 6

Medtr5g078930 ----------------------------------------------------MCMENSRI 8

Medtr5g079120 MAREDNRVAVAIGQACCRQKLLAQQMNALVPQAIENQKQFKVSRVLGKRIRKVRMENKRG 60

MtLAP1.R2R3SG6.antho.A ------------------------------------------------------MENTGG 6

Medtr8g060940 ------------------------------------------------------MENTGG 6

Medtr7g017260 ------------------------------------MKLIKERVYEDIKGVGMDMDNTIG 24

Medtr5g078800 ------------------------------------------------------MEKTRG 6

Medtr5g078860 ------------------------------------------------------MEKARG 6

**Psote05G0098700.1 ------------------------------------------------------------ 0**

EsMYBA1.R2R3SG6.antho.A ------------------------------------------------MKPDFSEMFKSG 12

Glyma.19G025000 ------------------------------------------------------MDGSAC 6

LhMYB6.R2R3SG6.antho.A ------------------------------------MSPFRVSATSSSFSQMSPSPVLRL 24

InMYB1.R2R3SG6.antho.A ----------------------------------------------MV---NSSARWSPR 11

Medtr5g079220 ------------------------------------MPLF-VCGQKQV---YMEKCKTRG 20

GhMYB10.R2R3SG6.antho.A ----------------------------------------------------MGAEARSG 8

GamMYB10.R2R3SG6.antho.A ------------------------------------------------------MERSSG 6

AmROSEA1.R2R3SG6.antho.A ----------------------------------------------------ME-KNCRG 7

VlMYBA2.R2R3SG6.antho.A -------------------------------------------------------MESLG 5

VvMYBA1.R2R3SG6.antho.A -------------------------------------------------------MESLG 5

StAN1.R2R3SG6.PP.A ---------------------------------------------MST---PMMCTFLGV 12

MrMYB1.R2R3SG6.antho.A -----------------------------------------------------M-EGSLG 6

PhDPL.R2R3SG6.antho.A ---------------------------------------------MNT---SVF-TSSGV 11

PhPHZ.R2R3SG6.antho.A ---------------------------------------------MNT---TIP-KSSGL 11

PhAN2.R2R3SG6.antho.A ----------------------------------------------MS---TSN-ASTSG 10

NtAN2.R2R3SG6.antho.A ---------------------------------------------MNI---CTN-KSSSG 11

CsRUBY.R2R3SG6.antho.A -----------------------------------------------------M-ADSLG 6

AtMYB113.R2R3SG6.antho.A ----------------------------------------------------MG-ESPKG 7

AtMYB90/PAP2.R2R3SG6.antho.A ----------------------------------------------------ME-GSSKG 7

AtMYB75/PAP1.R2R3SG6.antho.A ----------------------------------------------------ME-GSSKG 7

AtMYB114.R2R3SG6.antho.A ----------------------------------------------------ME-GSSKG 7

BoPAP1.R2R3SG6.antho.A ----------------------------------------------------ME-DSSKG 7

______________R2__________________________________________

PpMYB10.R2R3SG6.antho.A VRKGAWTREEDDLLRQCIENQGEGKWHQVPYKA--GLNRCRKSCRLRWVNYLKPNIKKGE 65

MdMYB1.R2R3SG6.antho.A VRKGAWTREEDNLLRQCVEIHGEGKWNQVSYKA--GLNRCRKSCRQRWLNYLKPNIKRGD 67

MdMYB10.R2R3SG6.antho.A VRKGAWTREEDNLLRQCVEIHGEGKWNQVSYKA--GLNRCRKSCRLRWLNYLKPNIKRGD 67

PcMYB10.R2R3SG6.antho.A VRKGAWTREEDNLLRQCIEIHGEGKWNQVSYKA--GLNRCRKSCRQRWLNYLKPNIKRGD 67

PyMYB10.R2R3SG6.antho.A VRKGAWTREEDNLLRQCIEIHGEGKWNQVSYKA--GLNRCRKSCRQRWLNYLKPNIKRGD 67

**Psote05G0036700.1 VRKGAWSKLEDNLLKASVQLYGEGKWHLIPQRA--GLNRCRKSCRLRWLNYLKPNIKRGD 70**

**Psote05G0036500.1 VRKGAWNQFEDDLLRDCVKLYGEGKWHLVPQRA--GLNRCRKSCRLRWLNYLKPNIKRGD 64**

**Psote05G0036600.1 VRKGAWNQFEDDLLKDCVNLYGEGKWHLVPQRA--GLNRCRKSCRLRWLNYLKPNIKRGD 64**

**Psote05G0036600.1_FP15 VRKGAWNQFEDDLLKDCVNLYGEGKWHLVPQRA--GLNRCRKSCRLRWLNYLKPNIKRGD 64**

Glyma.18G261700 VRKGAWTKCEDDLLRACVQLYGEGKWHLVPQRA--GLNRCRKSRRLRWLNYLKPNIKRGD 64

Glyma.09G235300 VRKGAWSKCEDDLLRACVQLYGEGKWHLVPQRA--GLNRCRKSCRLRWLNYLKPNIKRGD 64

Glyma.18G262000 VRKGAWSQFEDDLLRDCVNLHGEGKWHLVPQRA--GLNRCRKSCRLRWLNYLKPNIKRGD 64

Glyma.09G234900 VRKGAWSQIEDNLLRDCVNLHGEGKWHLVPKRA--GLNRCRKSCRLRWLNYLKPNIKRGD 64

Glyma.09G235100 VRKGTWSQIEDDLLKACVQLYGEGNWHLVPKRA--GLNRCRKSCRLRWLNYLKPNIKRGD 64

StMTF1.R2R3SG6.PP.A VRKGSWTDEEDFLLRKCIDKYGEGKWHLVPARA--GLNRCRKSCRLRWLNYLRPHIKRGD 68

LeANT1.R2R3SG6.antho.A VRKGSWTDEEDFLLRKCIDKYGEGKWHLVPIRA--GLNRCRKSCRLRWLNYLRPHIKRGD 68

Medtr5g079290 VRKGAWTYEEDKLLKACIQKYGEGKWHLVPQRA--GLNRCRKSCRLRWLNYLTPNIKRES 74

Medtr5g078950 VKRGAWTYEEDKLLKACINKYGEGKWHFVPQRA--GLSRCRKSCRLRWLNYLSPNIKRES 64

Medtr5g078910 LRKGAWTYEEDKLLKACIQKYGEGKWHLVSQRA--GLNRCRKSCRLRWVNYLCPNIKRES 64

Medtr5g078930 KRKRTWTSEEDKLLKAYINKYGEGKWHLIPKRA--GLNRCRKSCRLRWLNYLNPTINREI 66

Medtr5g079120 LRKGAWTYEEDNLLKAYIHKYGEGKWHLIPKRT--GLNRCRKSCRLRWVNYLNPNINRES 118

MtLAP1.R2R3SG6.antho.A VRKGAWTYKEDELLKACINTYGEGKWNLVPQRS--GLNRCRKSCRLRWLNYLSPNINRGR 64

Medtr8g060940 VRKGAWTYKEDELLKACINTYGEGKWNLVPQRS--GLNRCRKSCRLRWLNYLSPNINRGR 64

Medtr7g017260 VKKGAWTYEEDNLLKAYINKYGEGKWHLIPQRAGSGLNRCRKSCRLRWINYLKPNINRKS 84

Medtr5g078800 LRKGAWTYEEDNLLKDCIQKYGEGKWHLVPKRA--GLNRCRKSCRLRWVNYLNPAINRES 64

Medtr5g078860 LRKGAWTYEEDNLLKACIHKYGEGKWHLVPKRT--GLNRCRKSCRLRWLNYLNPAINRES 64

**Psote05G0098700.1 MGGVAWTEEEDHLLKKCIQQYGEGKWHRVPLLA--GLNRCRKSCRLRWLNYLRPNIKRGN 58**

EsMYBA1.R2R3SG6.antho.A VRKGAWTKEEDEVLKICVEKYGVGNWHRIPQRA--GLNRCRKSCRMRWLNYLNPSINRGV 70

Glyma.19G025000 VRKGLWSEVEDTLLRTCVRQYGEGQWHLVPTRA--GLNRCRKSCRLRWLNYLKPNIKRGE 64

LhMYB6.R2R3SG6.antho.A VRKGAWTQVEDDLLKRCIERHGVVRWSRVPQLA--GLNRCRKSCRLRWLNYLDPRIRRGQ 82

InMYB1.R2R3SG6.antho.A VRKGAWSEEEDDLLRKCIQKFGEGKWHLVPFRA--GLNRCRKSCRLRWLNYLHPDIKRGH 69

Medtr5g079220 VRKGAWTYEEDKLLKACMQKYGEGKWHLVPQRA--GLNRCRKSCRLRWLNYLNPTINRES 78

GhMYB10.R2R3SG6.antho.A LRKGAWTAEEDMLLKNCIERYGEGKWHLVPLKA--GLNRCRKSCRLRWLNYLRPNIKRGD 66

GamMYB10.R2R3SG6.antho.A IRKGTWTVEEDKLLRMCVEKYGEGKWHQIPKKA--GLNRCRKSCRLRWLNYLKPNIKRGD 64

AmROSEA1.R2R3SG6.antho.A VRKGTWTKEEDTLLRQCIEEYGEGKWHQVPHRA--GLNRCRKSCRLRWLNYLRPNIKRGR 65

VlMYBA2.R2R3SG6.antho.A VRKGAWIQEEDVLLRKCIEKYGEGKWHLVPLRA--GLNRCRKSCRLRWLNYLKPDIKRGE 63

VvMYBA1.R2R3SG6.antho.A VRKGAWIQEEDVLLRKCIEKYGEGKWHLVPLRA--GLNRCRKSCRLRWLNYLKPDIKRGE 63

StAN1.R2R3SG6.PP.A IRKGSWTEEEDILLRKCIDKYGEGKWHLVPTRA--GLNRCRKSCRLRWLNYLRPHIKRGD 70

MrMYB1.R2R3SG6.antho.A VRKGAWTVEEDTLLKLYIEKYGEGKWHQVPPRA--GLNRCRKSCRLRWLNYLKPNIKRGE 64

PhDPL.R2R3SG6.antho.A LRKGAWAEEEDILLRKCIEKYGEGKWHQVPVRA--GLNRCRKSCRLRWLNYLRPHIKRGD 69

PhPHZ.R2R3SG6.antho.A VRKGAWTEEEDVLLRKCIEKFGEGKWHQVPVRA--GLNRCRKSCRLRWLNYLRPHIKRGD 69

PhAN2.R2R3SG6.antho.A VRKGAWTEEEDLLLRECIDKYGEGKWHLVPVRA--GLNRCRKSCRLRWLNYLRPHIKRGD 68

NtAN2.R2R3SG6.antho.A VKKGAWTEEEDVLLKKCIEKYGEGKWHQVPLRA--GLNRCRKSCRLRWLNYLRPHIKRGD 69

CsRUBY.R2R3SG6.antho.A VRKGAWTGEEDDLLRKCIEKYGEAKWHQVPLRA--GLHRCRKSCRLRWLNYLNPNIKRGE 64

AtMYB113.R2R3SG6.antho.A LRKGTWTTEEDILLRQCIDKYGEGKWHRVPLRT--GLNRCRKSCRLRWLNYLKPSIKRGK 65

AtMYB90/PAP2.R2R3SG6.antho.A LRKGAWTAEEDSLLRLCIDKYGEGKWHQVPLRA--GLNRCRKSCRLRWLNYLKPSIKRGR 65

AtMYB75/PAP1.R2R3SG6.antho.A LRKGAWTTEEDSLLRQCINKYGEGKWHQVPVRA--GLNRCRKSCRLRWLNYLKPSIKRGK 65

AtMYB114.R2R3SG6.antho.A LRKGAWTAEEDSLLRQCIGKYGEGKWHQVPLRA--GLNRCRKSCRLRWLNYLKPSIKRGK 65

BoPAP1.R2R3SG6.antho.A LTKGAWTAEEDSLLRRCIDKYGEGKWHQVPLRA--GLNRCRKSCRLRWLNYLKPTIKRGK 65

* ** :*: : * .* : : ** ***** * **:*** * *.:

_________R3___________________|_helix 3|______

PpMYB10.R2R3SG6.antho.A FAEDEVDLIIRLHKLLGNRWSLIARRLPGRTANNVKNYWNTRSRTDYCMKKIKD------ 119

MdMYB1.R2R3SG6.antho.A FKEDEVDLIIRLHRLLGNRWSLIARRLPGRTANAVKNYWNTRLRIDSRMKTVKN------ 121

MdMYB10.R2R3SG6.antho.A FKEDEVDLIIRLHRLLGNRWSLIARRLPGRTANAVKNYWNTRLRIDSRMKTVKN------ 121

PcMYB10.R2R3SG6.antho.A FKEDEVDLILRLHRLLGNRWSLIARRLPGRTANDVKNYWYTRLRIDSRMKTVKN------ 121

PyMYB10.R2R3SG6.antho.A FKEDEVDLILRLHKLLGNRWSLIARRLPGRTANDVKNYWNTRLRIDSRMKTLKN------ 121

**Psote05G0036700.1 FSEDEIDLMIRMHKLLGNRWSLIAGRLPGRTSNDVKNYWNTYARRKLLSHKKDNIVKK-- 128**

**Psote05G0036500.1 FSEDEVDLMIRMHKLLGNRWSLIAGRLPGRTSNDVKNYWNTYMRRKVQS---DKNVKK-- 119**

**Psote05G0036600.1 FSEDEVDLMIRMHKLLGNRWSLIAGRLPGRTSNDVKNYWNTYMRRKVQS---DKNVKK-- 119**

**Psote05G0036600.1_FP15 FSEDEVDLMIRMHKLLGNRWSLIAGRLPGRTSNDVKNYWNSYMRRKVHSDKKEKNVKK-- 122**

Glyma.18G261700 LSEDEVDMMIRMHKLLGNRWSLIAGRLPRRTSNDVKNYWNTYMRRKVYSHKKDNNVEK-- 122

Glyma.09G235300 FSEDEVDMMIRMHKLLGNRWSLIAGRLPGRTSNDVKNYWNTYMRRKVYSHKKDNNVEK-- 122

Glyma.18G262000 FNEDEVDLMIRLHKLLGNRWSLIAGRLPGRTSNDVKNYWNAYMRRKVHSHNKDNKIKK-- 122

Glyma.09G234900 FSEDEVDLMIRLHKLLGNRWSLIAGRLPGRTSNDVKNYWNTYMRRKVHSHKKDNNIEK-- 122

Glyma.09G235100 FSEDEIDMMIRLHKLLGNRWSLIAGRLPGRTSNDVKNYWNTYARRKLHSHKKDNNIEK-- 122

StMTF1.R2R3SG6.PP.A FAPDEVDLILRLHKLLGNRWSLIAGRLPGRTANDVKNYWNTNLLRSKVNITTKFVPHEKI 128

LeANT1.R2R3SG6.antho.A FEQDEVDLILRLHKLLGNRWSLIAGRLPGRTANDVKNYWNTNLLRKL--NTTKIVPREKI 126

Medtr5g079290 FAEDEVDMMLRLHKLLGNRWSLIAARLPGRTANDVKNYWHTHLRKKMVSRTLE-EKKE-- 131

Medtr5g078950 FAEDEVDLILRLQKLLGNRWSLIAARLPGRTANDVKNYWHTNLRKKLALE-KEKKEKE-- 121

Medtr5g078910 FAEDEVDMILRLHKLLGNRWSLIAARLPGRTANDVKNFWHTHLRKKVVSETKEKKEKE-- 122

Medtr5g078930 FSEDEADMILRLHNLLGNRWTLIAARLQGRTANDVKNYWNTHLKNKDVSRAKEKKEKD-- 124

Medtr5g079120 ITEDEADMIIRLHNLLGNRWSLIAARLPSRTANDVKNYWNTHLRKKVLSESVEKNEKE-- 176

MtLAP1.R2R3SG6.antho.A FSEDEEDLILRLHKLLGNRWSLIAGRLPGRTANDVKNYWHTNLAKKVVSEKEEEKEND-- 122

Medtr8g060940 FSEDEEDLILRLHKLLGNRWSLIAGRLPGRTANDVKNYWHTNLAKKVVSEKEEEKEND-- 122

Medtr7g017260 FSEDEVDMILRLHKLLGNRWSLIAGRLPGRTANSVKNYWNTHLLKKVVSKQEEE--KE-- 140

Medtr5g078800 FSHDEIDMILRLHKLLGNRWSLIAARLPGRTANDVKNYWHTNLRKKVIPRKEENEEKEK- 123

Medtr5g078860 FAEDEIDMILRLHKLLGNKWSLIAARLPGRTANDVKNYWHTNLRKKVIPRKEEKEEKEK- 123

**Psote05G0098700.1 FAEEEVEMIIKLHKLLGNRWSLIAGRLPGRTANDVKNYWNCHLSKKLNALEAEDRQ---- 114**

EsMYBA1.R2R3SG6.antho.A FREDEIDLMLKMHKLLGNRWSLIAGRLPGRTANDVKNFWNTQLRHKSVLNNKDK------ 124

Glyma.19G025000 FTEDEVDLMQRLHNLLGNRWSLIAGRLPGRTPNDVKNYWNTYIRRKVSSSHKVVINEK-- 122

LhMYB6.R2R3SG6.antho.A FEEDEDDLIIRLHKLLGNRWSLIAGRLPGRTANDVKNYWNSHLSKKLIPQEKKVRACP-- 140

InMYB1.R2R3SG6.antho.A FSLEEADLILRLHKLLGNRWSLIAGRIPGRTANDVKNYWHSHLKKKVVGMHMASSNSSRQ 129

Medtr5g079220 FSEDEVDMILRLHKLLGNRWSLIAARLPGRTANDVKNYWHTHLRKKMVSRKEEKKENE-- 136

GhMYB10.R2R3SG6.antho.A FGEDEIDLIIRLHKLLGNRWSLIAGRIPGRTANDVKNWWNTHLRSRHQQQQKVHQE---- 122

GamMYB10.R2R3SG6.antho.A FLADEVDLMLKLHKLLGNRWSLIAGRLPGRTANDVKNFWNTHLKKRTVSPPEDEENLKS- 123

AmROSEA1.R2R3SG6.antho.A FSRDEVDLIVRLHKLLGNKWSLIAGRIPGRTANDVKNFWNTHVGKNLGEDGERCRKNV-- 123

VlMYBA2.R2R3SG6.antho.A FALDEVDLMIRLHNLLGNRWSLIAGRLPGRTANDVKNYWHGHHLKKKVQFQEEGR----- 118

VvMYBA1.R2R3SG6.antho.A FALDEVDLMIRLHNLLGNRWSLIAGRLPGRTANDVKNYWHSHHFKKEVQFQEEGR----- 118

StAN1.R2R3SG6.PP.A FEPDEVDLILRLHKLLGNRWSLIAGRLPGRTANDVKNYWNTNLLRKLNTSTKFAPQPQEG 130

MrMYB1.R2R3SG6.antho.A FKADEVDLMIRLHKLLGNRWSMIAGRLPGRTANDVKNYWNTHLRKNAISRIKDG------ 118

PhDPL.R2R3SG6.antho.A FSPEEVDLILRLHKLLGNRWSLIAGRLPGRTANDVKNYWNTHLLRRSNFAPPPQHERK-- 127

PhPHZ.R2R3SG6.antho.A FSEDEVDLIFRLHKLLGNRWSLIAGRLPGRTANDVKNYWNTHLQRKLIAPARQEI----- 124

PhAN2.R2R3SG6.antho.A FSLDEVDLILRLHKLLGNRWSLIAGRLPGRTANDVKNYWNTHLRKKLIAPHDQKQESK-- 126

NtAN2.R2R3SG6.antho.A FSFDEVDLILRLHKLLGNRWSLIAGRLPGRTANDVKNYWNSHLRKKLIAPHDQ------- 122

CsRUBY.R2R3SG6.antho.A FAADEVDLILRLHKLLGNRWSLIVGRLPGRTANDVKNFWNTHLRKKVD-KCCKNNKEM-- 121

AtMYB113.R2R3SG6.antho.A LCSDEVDLVLRLHKLLGNRWSLIAGRLPGRTANDVKNYWNTHLSKKHDERCCKTKMINK- 124

AtMYB90/PAP2.R2R3SG6.antho.A LSNDEVDLLLRLHKLLGNRWSLIAGRLPGRTANDVKNYWNTHLSKKHESSCCKSKMKKK- 124

AtMYB75/PAP1.R2R3SG6.antho.A LSSDEVDLLLRLHRLLGNRWSLIAGRLPGRTANDVKNYWNTHLSKKHE-PCCKIKMKKR- 123

AtMYB114.R2R3SG6.antho.A FSSDEVDLLLRLHKLLGNRWSLIAGRLPGRTANDVKNYWNTHLSKKHE-PCCKTKIKRI- 123

BoPAP1.R2R3SG6.antho.A LSSDEVDLLLRLHKLLGNRWSLIAGRLPGRTANDIKNYWNTHLSKKHE-PCCKTKMKKR- 123

: :* ::: :::.****:*::*. *: ** * :**:*

[D/E]L_x2_[R/K]_x3_L_x6_L_x3_R [A/S/G]NDV

PpMYB10.R2R3SG6.antho.A ------------KSQETIK-TIIRPQPRRFTKSSN----CLSFKEPILDH------TQ-- 154

MdMYB1.R2R3SG6.antho.A ------------KSQEMRKTNVIRPQPQKFNRSSY----YLSSKEPILDH------IQ-- 157

MdMYB10.R2R3SG6.antho.A ------------KSQEMRETNVIRPQPQKFNRSSY----YLSSKEPILDH------IQ-- 157

PcMYB10.R2R3SG6.antho.A ------------KSQETRKTNVIRPQPQKFIKSSY----YLSSKEPILEH------IQ-- 157

PyMYB10.R2R3SG6.antho.A ------------KSQETRKTNVIRPQPQKFIKSSY----YLSSKEPILEH------IQ-- 157

**Psote05G0036700.1 --------HD--AESTEKPHQVIKPVPRTLS-TSP----CLHRNVVNCS--KVGISE--- 168**

**Psote05G0036500.1 --------QE--VEANVKPHQVIKPIPRAFSKPST----CLQGKLLSNNF-IVDVNE--- 161**

**Psote05G0036600.1 --------QE--VEANVKPHQVIKPIPRAFSKPST----CLQEKLVSSS--IVGVSE--- 160**

**Psote05G0036600.1_FP15 --------QE--AEANVKPHQVIKPVPRAFSKPTT----CLQEKLVSSS--IVGVSE--- 163**

Glyma.18G261700 --------QAE-AKSTVKHHEVIKPVPRTLSKTSP----WLQGKFFNSS--KVGASG--- 164

Glyma.09G235300 --------QADEAKSTVQRHQVIKPIPRALTKTAP----RLQGKLFNSS--KVGVSE--- 165

Glyma.18G262000 --------QE--TKSTVKPHEVIKPIPRVLTKTSP----WLQRKFINSP--KVGVSE--- 163

Glyma.09G234900 --------QADEAKPIVKHHEVIKPVPRTLSKTSP----WLQGKFVNSS--KVGVSE--- 165

Glyma.09G235100 --------QAR-AKTTVKPHEVIKPVPRALTKTSP----RLQGKFINSS--EVGVSH--- 164

StMTF1.R2R3SG6.PP.A N---------NKCGEIT-KNEIIKPQPRKYFSSTKKNIT----------NNIVIVDKEE- 167

LeANT1.R2R3SG6.antho.A N---------NKCGEISTKIEIIKPQRRKYFSSTMKNVT----------NNNVILDEEE- 166

Medtr5g079290 -----------KPKETMKVHEIIKPKPRTFSTHPP----SLNWKHNINVTPIVAV----- 171

Medtr5g078950 -----------KHKETMKTHEVIKPQPRIFSTHSP----WLNKKQNNFVTQPVLA----- 161

Medtr5g078910 -----------KPKETMKAHEVIKPQPRIFSTHSS----WLNRKHVNFVSQPMLA----- 162

Medtr5g078930 -----------NPKEIIKAHEVIKPRPLTFSTHSP----WLNGKH-NFVTHPILG----- 163

Medtr5g079120 -----------RPKETMKAHEVIIPRPITLSTHS------------SFMTQPILN----- 208

MtLAP1.R2R3SG6.antho.A -----------KPKETMKAHEVIKPRPITLSSHSN----WLKGKNS-------------- 153

Medtr8g060940 -----------KPKETMKAHEVIKPRPITLSSHSN----WLKGKNS-------------- 153

Medtr7g017260 -----------KPMETMKAHQVIKPRPITFSTQSS----WLNVKHNNFVTQPLLA----- 180

Medtr5g078800 -----------SKESMIKSHEVIKPRPRTFSTHSL----WLKKKRNFVSD------GSAT 162

Medtr5g078860 -----------SKESMIKSHEVIKPRPRTFSTHSL----WLKKKHNFVSN------GSAT 162

**Psote05G0098700.1 ---------------ITRDVEVIRPQARNIGSSSV----KRRGQGESP-----------T 144**

EsMYBA1.R2R3SG6.antho.A -----------ERILPPKKVEVIKPHPRIFKPVPT----RLT--GEPA------------ 155

Glyma.19G025000 -----------QKKTTVKPHVVIKPKARTFSRPSPSGLRG-----SSV------LREEGG 160

LhMYB6.R2R3SG6.antho.A -----------------CIAAPTRPQPRKCSIKTKTSVDDQQVNMSEL---IPQKKKVRA 180

InMYB1.R2R3SG6.antho.A DNNW---DDEKGKAPQIKENILFRPRPRRFFRTSLSSPALSTLTGKAK---AVAYDAPPP 183

Medtr5g079220 -----------KPKESMQTHEVIKPQPRTFSSHSP----WLNGKYNNFVTPIVTVSTNDG 181

GhMYB10.R2R3SG6.antho.A -----------DELSQDTTVAIIKPQPRTFSKTLN----WFGNRQSVKDH----VDINII 163

GamMYB10.R2R3SG6.antho.A -------P---TPQKIVTRGNIFKPRPRKFSNCSC----PFDASR-KK-----------S 157

AmROSEA1.R2R3SG6.antho.A -----------MNTKTIKLTNIVRPRARTFTGLHV---TWPREVGKTDEF----SNVRLT 165

VlMYBA2.R2R3SG6.antho.A -----------DKPQTHSKTKAIKPHPHKFSKALP----KFELKTTAVDT----FDTQVS 159

VvMYBA1.R2R3SG6.antho.A -----------DKPQTHSKTKAIKPHPHKFSKALP----RFELKTTAVDT----FDTQVS 159

StAN1.R2R3SG6.PP.A INTSTIAPQPQEGIKYGQANAIIRPQPQKFTSSMKINVSWCNNNSMVN------------ 178

MrMYB1.R2R3SG6.antho.A -------G---EKAQQTSKVNIIKPRPRTFAKNLT----WFGGKPTIM-A----ASFQPK 159

PhDPL.R2R3SG6.antho.A ------CT---KAVKIMAKNVIIRPQPRNLSKLAKNNVSNY--STIHK---------DE- 166

PhPHZ.R2R3SG6.antho.A ------RK---CRALKITENNIVRPRPRTFSNSAQNISWCSNKS-ITNST----IDTDG- 169

PhAN2.R2R3SG6.antho.A -----------NKAVKITENNIIKPRPRTFSRPAMNNFPCWNGKSCNKNT----IDKNEG 171

NtAN2.R2R3SG6.antho.A -------K---ESKQKAKKITIFRPRPRTFSKTNT----CVKSNTNTV------DKDIEG 162

CsRUBY.R2R3SG6.antho.A -------K---AKAEKVEKINIIKPQPRTFAKNSQ----WLKGKGMTS------NNLQLG 161

AtMYB113.R2R3SG6.antho.A ----NITS---HPTSSAQKIDVLKPRPRSFSDKNS----CNDVNILPK------VDVVPL 167

AtMYB90/PAP2.R2R3SG6.antho.A ----NIIS---PPTTPVQKIGVFKPRPRSFSVNNG----CSHLNGLPE------VDLIPS 167

AtMYB75/PAP1.R2R3SG6.antho.A ----DI**T**P---IPT**T**PALKNNVYKPRPRSFTVNND----CNHLNAPPK------VDVNPP 166

AtMYB114.R2R3SG6.antho.A ----NIIT---PPNTPAQKVDIF------------------------------------- 139

BoPAP1.R2R3SG6.antho.A ----NVTF---SSTTPAQKIDVFKPRPRLFTVSNG----CSHLHGLPE------VDVVPP 166

KPRPR[S/T]F

PpMYB10.R2R3SG6.antho.A ------------------------------------------------------------ 154

MdMYB1.R2R3SG6.antho.A ------------------------------------------------------------ 157

MdMYB10.R2R3SG6.antho.A ------------------------------------------------------------ 157

PcMYB10.R2R3SG6.antho.A ------------------------------------------------------------ 157

PyMYB10.R2R3SG6.antho.A ------------------------------------------------------------ 157

**Psote05G0036700.1 ------------------------------------------------------------ 168**

**Psote05G0036500.1 ------------------------------------------------------------ 161**

**Psote05G0036600.1 ------------------------------------------------------------ 160**

**Psote05G0036600.1_FP15 ------------------------------------------------------------ 163**

Glyma.18G261700 ------------------------------------------------------------ 164

Glyma.09G235300 ------------------------------------------------------------ 165

Glyma.18G262000 ------------------------------------------------------------ 163

Glyma.09G234900 ------------------------------------------------------------ 165

Glyma.09G235100 ------------------------------------------------------------ 164

StMTF1.R2R3SG6.PP.A ------------------------------------------------------------ 167

LeANT1.R2R3SG6.antho.A ------------------------------------------------------------ 166

Medtr5g079290 ----S------------------------------------------TQHGEVSPNRDNK 185

Medtr5g078950 ----SNKDGNVPRDSNASL----------------------------------------- 176

Medtr5g078910 ----SNKDGNVSTKHVNFVSQPMLASNKDGNVSTKHVNFVSQPMLASNKDGNVSKDRDNR 218

Medtr5g078930 ---------KVPRYHDASS----------------------------------------- 173

Medtr5g079120 ----SNNDSKILMDRDGSS----------------------------------------- 223

MtLAP1.R2R3SG6.antho.A ----------IPRDLDY-S----------------------------------------- 161

Medtr8g060940 ----------IPRDLDY-S----------------------------------------- 161

Medtr7g017260 ----SNNDGCFPRDRDDKM----------------------------------------- 195

Medtr5g078800 KLVISSEDGNVPRECD-------------------------------------------- 178

Medtr5g078860 KLVISSEDGNVPRECDKTTLPNLIDSSS------SHSPWL-----KKN----HSLPTTLM 207

**Psote05G0098700.1 DKGVQQ------------------------------------------------------ 150**

EsMYBA1.R2R3SG6.antho.A ------------------------------------------------------------ 155

Glyma.19G025000 ESGA-------------------------------------------------------- 164

LhMYB6.R2R3SG6.antho.A CRIIAAPTRPQPRKCSIE------------------------------------------ 198

InMYB1.R2R3SG6.antho.A PH----------HHQLQA-QPEAT-----------------------------SPPADLL 203

Medtr5g079220 NVAKDS------------------------------------------------------ 187

GhMYB10.R2R3SG6.antho.A KSSSASDTN-----NISA-PPELI-----------------------------ASPKI-- 186

GamMYB10.R2R3SG6.antho.A DIGINSLQS--------------------------------------------------- 166

AmROSEA1.R2R3SG6.antho.A TDEIP------------------------------------------------------- 170

VlMYBA2.R2R3SG6.antho.A TSSKPSSTSPQPNDDIIW-WESLL-----------------------------AEHAQMD 189

VvMYBA1.R2R3SG6.antho.A TSRKPSSTSPQPNDDIIW-WESLL-----------------------------AEHAQMD 189

StAN1.R2R3SG6.PP.A ------------------------------------------------------------ 178

MrMYB1.R2R3SG6.antho.A DNVI-------------------------------------------------------- 163

PhDPL.R2R3SG6.antho.A ------------------------------------------------------------ 166

PhPHZ.R2R3SG6.antho.A ------------------------------------------------------------ 169

PhAN2.R2R3SG6.antho.A DTEI-------------------------------------------------------- 175

NtAN2.R2R3SG6.antho.A SSEII------------------------------------------------------- 167

CsRUBY.R2R3SG6.antho.A DYNLG------------------------------------------------------- 166

AtMYB113.R2R3SG6.antho.A HLGLN------------------------------------------------------- 172

AtMYB90/PAP2.R2R3SG6.antho.A CLGLK------------------------------------------------------- 172

AtMYB75/PAP1.R2R3SG6.antho.A CLGLN------------------------------------------------------- 171

AtMYB114.R2R3SG6.antho.A ------------------------------------------------------------ 139

BoPAP1.R2R3SG6.antho.A CLGLN------------------------------------------------------- 171

PpMYB10.R2R3SG6.antho.A ---------------------------------------------RDWWETFLDDKDATE 169

MdMYB1.R2R3SG6.antho.A -------SAED--------------------LSTPPQTSSSTKNGNDWWETLLEGEDTFE 190

MdMYB10.R2R3SG6.antho.A -------SAED--------------------LSTPPQTSSSTKNGNDWWETLLEGEDTFE 190

PcMYB10.R2R3SG6.antho.A -------SAED--------------------LSTPSQTSSSTKNGNDWWETLFEGEDTFE 190

PyMYB10.R2R3SG6.antho.A -------SAED--------------------LSTPSQTSSPTKNGNDWWETLLEGEDTFE 190

**Psote05G0036700.1 ---------------------------------QGATS---SLVCGNWWNTLLHDKEN-- 190**

**Psote05G0036500.1 ---------------------------------AAGANAISSAVSENWWETLLEDNAG-- 186**

**Psote05G0036600.1 ---------------------------------AAGANAISSSESENWWETLLEDKED-- 185**

**Psote05G0036600.1_FP15 ---------------------------------AVGANAISSSESENWWETLLEDKED-- 188**

Glyma.18G261700 ---------------------------------EGATS---ISGSGNWWETLLDDKEDNE 188

Glyma.09G235300 ---------------------------------AAGAA---SSGSGNWWETLLENKKDNH 189

Glyma.18G262000 ---------------------------------EGATS------SENWWETLLADKEDNA 184

Glyma.09G234900 ---------------------------------EGATSI--SGSAGNWWETLLDDKEDNA 190

Glyma.09G235100 ---------------------------------EEGATS--ISGSGNWWETFLDDKEDIE 189

StMTF1.R2R3SG6.PP.A ----------HCKEII----------------SEKQTPDALMENVDQWWTNLLENCNDDV 201

LeANT1.R2R3SG6.antho.A ----------HCKEII----------------SEKQTPDASMDNVDPWWINLLENCNDDI 200

Medtr5g079290 EITDSNQIGRDIVGVSQPSL------------------GSAPIPCAMLWDSLLNLEEHKS 227

Medtr5g078950 GTTVSNQVGRDYVSSSQPSIDNVAILCS--------KKDNVAIPCAMLTDNLWNLGEPVD 228

Medtr5g078910 ETMVTYQNGRDCASASQPSI------------------DNVPIPCALSSDNLWDLGEQVG 260

Medtr5g078930 DTMVPDQIGRDCASDSQPRL------------------GNAPILCVQ------------- 202

Medtr5g079120 EIMVSNQIGRDCASASQQSL------------------GNVPVPCGMWSDSLWNLGEQVN 265

MtLAP1.R2R3SG6.antho.A ENMASNQIGRECASTSKPDL------------------GNAPIPCEMWCDSLWNLGEHVD 203

Medtr8g060940 ENMASNQIGRECASTSKPDL------------------GNAPIPCEMWCDSLWNLGEHVD 203

Medtr7g017260 TMVVPNQIGKDCASSSQPIL------------------GNVPIPCTMWSESLWNLGEQVD 237

Medtr5g078800 -------------KTTL------------PNIGDAQPCVGNVPLSTMWWESLLNVDEERN 213

Medtr5g078860 ASREDVNIPRECDKTTL------------PNVGDAQTCVGNDPSSTTWWESLLNMDEERS 255

**Psote05G0098700.1 ---------ESSMSSLTFD---A------DGQNHVLESQ----QDNIY-S-CLDQQAIVT 186**

EsMYBA1.R2R3SG6.antho.A ----------F---------------CNLQEQQQEEGNQHPVAEDTIWWEELLSHDKE-M 189

Glyma.19G025000 ---------KHCSTHHQA--------C------AASSEYINNWSTDQWWKTMMHDKGD-N 200

LhMYB6.R2R3SG6.antho.A ------TKTSVDEQQVNM---------------SESRPSADTANCAVWQDDL-GNVKE-M 235

InMYB1.R2R3SG6.antho.A MVFNVQQNNNSMATNF-----------------PAQTTAPPSHDGVKWWDLLYDDDHQ-G 245

Medtr5g079220 ----------EVDTILPI--------NG-DGDSAAQPYLENPTLSSMWWESLLNVSND-K 227

GhMYB10.R2R3SG6.antho.A ----LDDAINECRQKLF---------------DG-DEKEVDIDGHVRWSF---------- 216

GamMYB10.R2R3SG6.antho.A ----YQLSNN-SKSVI-------------SLQNHPLVPPISTEENPAWWETMLFEENL-E 207

AmROSEA1.R2R3SG6.antho.A ----------DCEKQTQF-------------YNDVASPQDEVEDCIQWWSKLLETTED-G 206

VlMYBA2.R2R3SG6.antho.A QETDFSASGEMLIASLRAE---ETATQKKGPMDGMIEQIQGGEGDIIWWESLLAEHAQ-M 245

VvMYBA1.R2R3SG6.antho.A QETDFSASGE-------------------------------------------------- 199

StAN1.R2R3SG6.PP.A -----------------------------------NEEASKDNNDMQWWANILENCND-I 202

MrMYB1.R2R3SG6.antho.A -----------------------------S---DLPPAPLPSENSVKWGENLFDDKEA-G 190

PhDPL.R2R3SG6.antho.A ----------HSKQKMFI-------------EKPTAAEVVSRDNNVEWWTNLLLDNCN-G 202

PhPHZ.R2R3SG6.antho.A -------SNNEC---IRI-------------NDKKPMAEVSRDDGVQWWTSLLANCNE-N 205

PhAN2.R2R3SG6.antho.A ---------------IKF-----------SDE--KQKPEESIDDGLQWWANLLANNIE-I 206

NtAN2.R2R3SG6.antho.A ----------------RF--------N----DNLKPTTEELTDDGIQWWADLLANNYN-N 198

CsRUBY.R2R3SG6.antho.A ---------K---QSTP------------SDHHHHHQQQQENETESVWWESFLFGDEL-D 201

AtMYB113.R2R3SG6.antho.A -------NNYVCESSIT---------CNKDEQKDKLININLLDGDNMWWESLLE-----A 211

AtMYB90/PAP2.R2R3SG6.antho.A -------KNNVCENSIT---------CNKDDEKDD-FVNNLMNGDNMWLENLLGENQE-A 214

AtMYB75/PAP1.R2R3SG6.antho.A -------INNVCDNSIIY--------NK-DKKKDQ-LVNNLIDGDNMWLEKFLEESQE-V 213

AtMYB114.R2R3SG6.antho.A ------------------------------------------------------------ 139

BoPAP1.R2R3SG6.antho.A ------NINNVCENSMTY--------CNKAGEKYE-LFSNLMDGENMWWESLLEESKQ-P 215

PpMYB10.R2R3SG6.antho.A R-A--TGSGLGLDEE------------LLASFWVDDDMPQSTRKCIN----FSE-GLIRG 209

MdMYB1.R2R3SG6.antho.A R-A--AYPSIELEEE------------LFTSFWFDDRLS--PRSCAN----FPE-GQSRS 228

MdMYB10.R2R3SG6.antho.A R-A--AYPSIELEEE------------LFTSFWFDDRLS--PRSCAN----FPE-GHSRS 228

PcMYB10.R2R3SG6.antho.A R-A--ACPSIELEEE------------LFTSFWFDDRLS--ARSCAN----FPEEGQSRS 229

PyMYB10.R2R3SG6.antho.A R-A--PCPSIELEEE------------LFTTFWFDDRLS--ARSCAN----FPEEGQSRS 229

**Psote05G0036700.1 ----NDSTCVFGKGDG------------VLDLSGE-DLTSF-------SCDFFNEDEIWT 226**

**Psote05G0036500.1 ----NIGTCFFSGEDA------------VLD--FL-P----------------------- 204**

**Psote05G0036600.1 -----NNTCFFGGEDG------------VVS--DQ-NLISI-------TCDFLTEAETWS 218**

**Psote05G0036600.1_FP15 -----NNTCFFGGEDG------------VVS--DQ-NLISI-------TCDFLTEAETWS 221**

Glyma.18G261700 G--NINNTCFFGGVES------------LTF-GMR-SLIQL-------LVTFLLKVKIGV 225

Glyma.09G235300 EGNIINNTCFFGGADG------------ELDLWDE-ELNPI-------ACDFLSEGENWS 229

Glyma.18G262000 VFNNNNNTCFFGGVHG------------ELNLWNE-ELTSI-------DFDFVTQGELGG 224

Glyma.09G234900 V--NNNNTCFFGGADG------------EFNLWSE-ELTSI-------DCDFVTQGESWS 228

Glyma.09G235100 E--GNNNKCFFGGEDG------------ALDLWGE-ELNSI-------ACDFLTQGETWS 227

StMTF1.R2R3SG6.PP.A EEE--EEEAVT------------NYEKTLTSLL---------NGEGNSMQQGQISHESWG 238

LeANT1.R2R3SG6.antho.A EED--E-EVVI------------NYEKTLTSLLHEEISPPLNIGEGNSMQQGQISHENWG 245

Medtr5g079290 SEK--IGSGSLLQEENF-IS----------------------------E-FPNVDDSFWD 255

Medtr5g078950 SEK--IGSCSSLQEEYF-SM----------------------------E-FSTIDYSFWD 256

Medtr5g078910 SEQ--IGSGSSIQE-NF-NM----------------------------E-FLDVDDSLWN 287

Medtr5g078930 ------CGCSSLQEQNYKVF----------------------------K-WL-------- 219

Medtr5g079120 IDK--IGSCSSLQEENNFNM----------------------------E-VPNVDDFFWD 294

MtLAP1.R2R3SG6.antho.A SEK--IGSCSSLQEENLMEF----------------------------P-NV-DDDSFWD 231

Medtr8g060940 SEK--IGSCSSLQEENLMEF----------------------------P-NV-DDDSFWD 231

Medtr7g017260 SEI--IGSSSSLQVENYEEF----------------------------S-IV-D--DFWD 263

Medtr5g078800 NEK--NGSCSLLQEENFT-----LEFSNVEEFFTN------------GS---TASDSFWD 251

Medtr5g078860 NEK--IDSSSLLPKENFT-----LEFSNVEDFFNN------------GP---TVSDSDWD 293

**Psote05G0098700.1 ELS--MDFQLEGF----------------E--------------AMMNG-EGSSSQWDWG 213**

EsMYBA1.R2R3SG6.antho.A NHG--TSVSFGREEVVSTT-----------NS-TE--------EERKAA-LFSDVDFEFQ 226

Glyma.19G025000 LDN--NQCLLGYQDEVGKLVKDNNCDENLASLTTQ------------EG-EFLIEGQNWS 245

LhMYB6.R2R3SG6.antho.A IEQ--LTEATI-P-------------------------SENTEGFAHEG-LMQDGVSLWD 266

InMYB1.R2R3SG6.antho.A LID--WT--------------------------------------------------TDD 253

Medtr5g079220 IGS--CS--LLLPEE--------YSKLNVENFL-------------------AEGPSTVG 256

GhMYB10.R2R3SG6.antho.A --------------------------------------TPADEEPLNIV-DQENGHDSLL 237

GamMYB10.R2R3SG6.antho.A ENK--LDTKANGWCE--------QDDQFLTSFFNG-EITQ--GTTVEGS-TKNDESGHWP 253

AmROSEA1.R2R3SG6.antho.A ELG--NLFEEAQQIG-------N------------------------------------- 220

VlMYBA2.R2R3SG6.antho.A DQE--TDFSASGE-------------MLIASLRTE-ETATQKKGPMD-G-MIEQIQGGEG 287

VvMYBA1.R2R3SG6.antho.A --------------------------MLIASLRTE-ETATQKKGPMD-G-MIEQIQGGEG 230

StAN1.R2R3SG6.PP.A GEG--EAERTL-PSC--KEI-NCNEIDKAPSLLHE---------GGNSM-QQGQGDGGWD 246

MrMYB1.R2R3SG6.antho.A DEI--GTYDVGGLNE-----------EPIATFRWA-EAAP--AETVGTP-LDEFGPSFWA 233

PhDPL.R2R3SG6.antho.A FEK--AA-----PES-------TSAVKNIENLLNEELLSTSINGGTNYP-MQETGDMGWS 247

PhPHZ.R2R3SG6.antho.A DEP--AVENMSY--------------DKLPSLLHE-EISPPMNGGISD--CMQEGQSGWD 246

PhAN2.R2R3SG6.antho.A EEL--VS-------------------CNSPTLLHE-ETAPSV--NAESS-LTQGGGSGLS 241

NtAN2.R2R3SG6.antho.A N-G--IE------EA-------D---NSSPTLLHE-EMPLLS------------------ 220

CsRUBY.R2R3SG6.antho.A QQG--ISSSLSRPEE-------E---STTANIFAE-KSPVVTKVTENRV-IEAGQSCPTD 247

AtMYB113.R2R3SG6.antho.A D-V--LG-----PEA--------------------------------------TETAKGV 225

AtMYB90/PAP2.R2R3SG6.antho.A D-A--IV-----PEA--------------------------------------TTAEHGA 228

AtMYB75/PAP1.R2R3SG6.antho.A D-I--LV-----PEA--------------------------------------TTTEKGD 227

AtMYB114.R2R3SG6.antho.A ------------------------------------------------------------ 139

BoPAP1.R2R3SG6.antho.A D-G--LV-----PKG--------------------------------------TATKKGA 229

PpMYB10.R2R3SG6.antho.A DFSFSV----------------DPW---NHSKEE---------------- 224

MdMYB1.R2R3SG6.antho.A EFSFST----------------DLW---NHSKEE---------------- 243

MdMYB10.R2R3SG6.antho.A EFSFST----------------DLW---NHSKEE---------------- 243

PcMYB10.R2R3SG6.antho.A EFSFSM----------------DLW---NHSKEE---------------- 244

PyMYB10.R2R3SG6.antho.A EFSFSM----------------DLW---NHSKEE---------------- 244

**Psote05G0036700.1 DVFLNLGN------------------------------------------ 234**

**Psote05G0036500.1 -------------------------------------------------- 204**

**Psote05G0036600.1 DLLLHIGD------------------------------------------ 226**

**Psote05G0036600.1_FP15 DLLLHIGE------------------------------------------ 229**

Glyma.18G261700 SFFLTYSRLMCVC---ECACVLGL----------CFHRLTSSSKIFKLP- 261

Glyma.09G235300 DFLLDL-------------------------------------------- 235

Glyma.18G262000 IVNFQNP------------------------------------------- 231

Glyma.09G234900 DFLLDLQG------------------------------------------ 236

Glyma.09G235100 DFLLDLGLGD---------------------------------------- 237

StMTF1.R2R3SG6.PP.A DFSLNLPPMQLGE-NDDFSAEIDLW---NLLD------------------ 266

LeANT1.R2R3SG6.antho.A EFSLNLPPMQQGVQNDDFSAEIDLW---NLLD------------------ 274

Medtr5g079290 FNLCDFD---------------SL-------------------------- 264

Medtr5g078950 SNLCDFI---------------SL-------------------------- 265

Medtr5g078910 SNLCDFD---------------SLL---DLY------------------- 300

Medtr5g078930 -------------------------------------------------- 219

Medtr5g079120 FNLDDFD---------------FLM---NL-------------------- 306

MtLAP1.R2R3SG6.antho.A FNLCDLN---------------SLW---DLP------------------- 244

Medtr8g060940 FNLCDLN---------------SLW---DLP------------------- 244

Medtr7g017260 FNICDYD---------------SLW---DL-------------------- 275

Medtr5g078800 FNLCDIN---------------NSS---GVFN------------------ 265

Medtr5g078860 SNLYDFN---------------F-F---GVFN------------------ 306

**Psote05G0098700.1 DLLLDM----------------DLYK------------------------ 223**

EsMYBA1.R2R3SG6.antho.A DFS-DL----------------NFWNFE---------------------- 237

Glyma.19G025000 DFFLDTN----------------L-------------------------- 253

LhMYB6.R2R3SG6.antho.A NFIFDI----------------QLS---S--------------------- 276

InMYB1.R2R3SG6.antho.A DFPIDVD----------------LL---KLLDTTI--------------- 269

Medtr5g079220 DFSWDS----------------TICEFDSLLDDILN-------------- 276

GhMYB10.R2R3SG6.antho.A DFPIDE----------------VVW---DLLN------------------ 250

GamMYB10.R2R3SG6.antho.A DLGFDE----------------AAW---SLFSPEQMANMSPSNTMFDMQM 284

AmROSEA1.R2R3SG6.antho.A -------------------------------------------------- 220

VlMYBA2.R2R3SG6.antho.A DFPFDV----------------GFW---DTPNTQVNHLI----------- 307

VvMYBA1.R2R3SG6.antho.A DFPFDV----------------GFW---DTPNTQVNHLI----------- 250

StAN1.R2R3SG6.PP.A EFALD-----------------DIW---NLLN------------------ 258

MrMYB1.R2R3SG6.antho.A EFPSNL----------------DVW---DFLDP----------------- 247

PhDPL.R2R3SG6.antho.A DFSIDS----------------DLW---ELLLQ----------------- 261

PhPHZ.R2R3SG6.antho.A DFSVDID---------------HLW---NLLN------------------ 260

PhAN2.R2R3SG6.antho.A DFSVDID---------------DIW---DLVS------------------ 255

NtAN2.R2R3SG6.antho.A -------------------------------------------------- 220

CsRUBY.R2R3SG6.antho.A DFAFDA----------------ELW---DLLNAK---------------- 262

AtMYB113.R2R3SG6.antho.A TLPLDFE---------------QIW---ARFDEETLELN*---------- 246

AtMYB90/PAP2.R2R3SG6.antho.A TLAFDVE---------------QLW---SLFDGETVELD----------- 249

AtMYB75/PAP1.R2R3SG6.antho.A TLAFDVD---------------QLW---SLFDGETVKFD----------- 248

AtMYB114.R2R3SG6.antho.A -------------------------------------------------- 139

BoPAP1.R2R3SG6.antho.A TFAFDVE---------------QLW---NMLDGETVELD----------- 250

Supplementary Data 17. Amino acid alignment of R2R3 MYB TF Subgroup 6 orthologues. Amino acid variation of Psote05G0036600.1 (*PtMYB113b*) among Ma3 and FP15 are underlined. Red font: amino acid insertion; blue font: phosphorylation sites. Abbreviations: At: *Arabidopsis thaliana*, Bo: *Brassica oleracea*; Cs: *Citrus sinesisI*; Es: *Epimedium sagittatum*; Gam: *Garinia mangostana*; Gh: *Gerbera hybrida*; In: *Ipomoea nil*; Le: *Lycopersicum esculentum*; Lh: *Lilium spp*.; Md: *Malus domestica*; Mr: *Myrica rubra*; Mt: *Medicago truncatulaI*; Nt: *Nicotiana tabacum*; Pc: *Pyrus communisis;* Ph: *Petunia hydrida*; Pp: *Prunus persica*; Py: *Pyrus pyrifoliaI;* St: *Solanum tuberosum*; Vl: *Vitis spp*.; Vv: *Vitis vinifera*.
