# Supplementary material for: A genomic toolkit for winged bean Psophocarpus tetragonolobus
Source: Nat Commun. 2024 Mar 1;15:1901. doi: 10.1038/s41467-024-45048-x (PMC10907731; doi:10.1038/s41467-024-45048-x)
Supplement: Supplementary file 23 — Reporting Summary [file 41467_2024_45048_MOESM23_ESM.pdf]

nature portfolio

Corresponding author(s): Sean Mayes

Last updated by author(s): Dec 4, 2023

Reporting Summary

Nature Portfolio wishes to improve the reproducibility of the work that we publish. This form provides structure for consistency and transparency in reporting. For further information on Nature Portfolio policies, see our Editorial Policies and the Editorial Policy Checklist.

Statistics

For all statistical analyses, confirm that the following items are present in the figure legend, table legend, main text, or Methods section.

n/a	Confirmed	
☐	☒	The exact sample size (*n*) for each experimental group/condition, given as a discrete number and unit of measurement
☐	☒	A statement on whether measurements were taken from distinct samples or whether the same sample was measured repeatedly
☐	☒	The statistical test(s) used AND whether they are one- or two-sided<br>*Only common tests should be described solely by name; describe more complex techniques in the Methods section.*
☒	☐	A description of all covariates tested
☒	☐	A description of any assumptions or corrections, such as tests of normality and adjustment for multiple comparisons
☐	☒	A full description of the statistical parameters including central tendency (e.g. means) or other basic estimates (e.g. regression coefficient) AND variation (e.g. standard deviation) or associated estimates of uncertainty (e.g. confidence intervals)
☐	☒	For null hypothesis testing, the test statistic (e.g. *F*, *t*, *r*) with confidence intervals, effect sizes, degrees of freedom and *P* value noted<br>*Give P values as exact values whenever suitable.*
☐	☒	For Bayesian analysis, information on the choice of priors and Markov chain Monte Carlo settings
☒	☐	For hierarchical and complex designs, identification of the appropriate level for tests and full reporting of outcomes
☒	☐	Estimates of effect sizes (e.g. Cohen's *d*, Pearson's *r*), indicating how they were calculated

Our web collection on statistics for biologists contains articles on many of the points above.

Software and code

Policy information about availability of computer code

Data collection	Sequencing data was generated by an Illumina NextGen500 sequencer using inbuilt software for base quality scores, BioNano data was generated on the inbuilt software for BioNano Saphyr system, Oxford Nanopore data was generated on inbuilt software of PromethION system, DArTseq reads were generated from inbuilt software for Illumina Hiseq 2500. RNA-seq data was generated using Illumina NovaSeq6000. RT-qPCR was conducted using Rotor-Gene Q Series Software. IDT PrimerQuest Tool for primer design. R-scripts used for analysis are all available as a pipeline
Data analysis	Microsoft Excel, Bionano Access: 1.3.0; Bionano Tools: 1.3.8041.8044; Bionano Solve: Solve3.3_10252018; RefAligner: 7915.7989rel; Canu (version 2.1.1); NECAT (v0.0.1 update20200803); quickmerge, Racon v1.5.0; Pilon v1.22; EDTA pipeline; Jellyfish; GenomeScope 2.0; Trinity v2.11.0; CD-HIT v4.8.1; CAP3; TransDecoder v5.7.1; MAKER2 v2.31.10; BUSCO v5.4.3; MCScanX; SynViio (https://synvisio.github.io/#/); eggNOG-mapper v2; clusterProfiler v4.2.2; OrthoFinder v2.5.4; CAFE5; DArT analytical pipeline; JoinMap v5; Beagle v5.4; fastSTRUCTURE; TASSEL v5; ape v5.7; ade4 v1.7; adegenet v2.1.10; poppr v2.9.4; dartR v2.9.7; MapQTL v6; MapChart v2.32; Phylogenomics pipeline (https://github.com/sumanthmutte/Phylogenomics); hisat2 v2.0.5; GATK v4.4.0.0; featureCounts 1.5.0-p3; DESeq2 v1.20.0; FastQC v0.12.1; Trimmomatic v0.39; BWA v0.7.15; Picard v1.96; vcftools v0.1.16; MEGA 11

For manuscripts utilizing custom algorithms or software that are central to the research but not yet described in published literature, software must be made available to editors and reviewers. We strongly encourage code deposition in a community repository (e.g. GitHub). See the Nature Portfolio guidelines for submitting code & software for further information.

Data

Policy information about availability of data

All manuscripts must include a data availability statement. This statement should provide the following information, where applicable:

- Accession codes, unique identifiers, or web links for publicly available datasets
- A description of any restrictions on data availability
- For clinical datasets or third party data, please ensure that the statement adheres to our policy

The reference genome sequence and annotation files are available at ORCAE, (https://bioinformatics.psb.ugent.be/orcae/aocc/overview/Psote) and Figshare (https://doi.org/ 10.6084/m9.figshare.19196255). Raw reads for genome assembly were deposited at the NCBI BioProject ID: PRJNA808222 with BioSample ID SAMN26037071. Raw reads for DArTseq of diversity panel and re-sequencing of two parental lines were deposited at BioProject ID: PRJNA1034490. RNA-sequencing reads of maturing pods were uploaded to GSE246575, (https://www.ncbi.nlm.nih.gov/geo/query/acc.cgi?acc=GSE246575).
Database used: Plant Transcription Factor Database (PlantTFDB, http://planttfdb.gao-lab.org/index.php); Phytozome database (https://phytozome-next.jgi.doe.gov/) of Phaseolus vulgaris v2.1,Vigna unguiculata v1.2, Medicago truncatula Mt4.0v1, Lotus japonicus v1, Glycine soja v1.1, Vitis vinifera v2.1; Legumepedia database (https://cegresources.icrisat.org/legumepedia/index.php) of Cicer arietinum v1, Cajanus cajan v1, Trifolium subterraneum v1, Glycine max Lee v2.

Research involving human participants, their data, or biological material

Policy information about studies with human participants or human data. See also policy information about sex, gender (identity/presentation), and sexual orientation and race, ethnicity and racism.

Reporting on sex and gender	n/a
Reporting on race, ethnicity, or other socially relevant groupings	n/a
Population characteristics	n/a
Recruitment	n/a
Ethics oversight	n/a

Note that full information on the approval of the study protocol must also be provided in the manuscript.

Field-specific reporting

Please select the one below that is the best fit for your research. If you are not sure, read the appropriate sections before making your selection.

☒ Life sciences ☐ Behavioural & social sciences ☐ Ecological, evolutionary & environmental sciences

For a reference copy of the document with all sections, see nature.com/documents/nr-reporting-summary-flat.pdf

Life sciences study design

All studies must disclose on these points even when the disclosure is negative.

Sample size	For genetic mapping, the population sizes were n=221 (XB population) and n=183 (XT population), which were sufficient for unequivocal marker ordering in the space genetic map. Both populations share a common parental line, Ma3.
Data exclusions	The Genotype-by-Sequencing data was screened according to standard approaches, with MAF (0.05) used to screen out rare alleles for the genetic diversity analysis and all markers with missing data removed for the mapping purpose. Phenotypic measurements were
Replication	For the QTL analysis (performed independently), single plant replications were used and the borders of the trial planted with additional F2 individuals to reduce border effects on traits. Parental lines (n=5 each) were also grown in the trials. Quantitative traits when possible, biological replicates were recorded; pod length is the average of first 15 fully developed pods per plant, branch length is the average of all branches longer than 10cm per plant. For the diversity analysis, a range of 'duplicates' samples from the breeding trials of EWS in the Philippines were included as they represented samples from the same parental accessions. However, it was unclear what an accession was composed of or what level of error or outcrossing occurred during the development of the material. Technical replicates (n=18) were run throughout the run for XB population seed protein profiling (SD = 1.156, SE = 0.273) whilst 5 biological replicates (and 3 technical reps of selected plants) of parental lines included. For protein content on developing seed stages, 3 biological replicates from different 3 plants were included.
Randomization	The F2 trait analysis was essentially randomised as seed from the F1 plant were mixed and planted. The seed protein profiling was conducted on entirely XB populations when the F3 lines had sufficient seed materials.

Blinding	As the individuals in the QTL analysis represented random F2 plants, there was no need for blinding. The lines subjected to seed protein content was solely based on material availability.

Reporting for specific materials, systems and methods

We require information from authors about some types of materials, experimental systems and methods used in many studies. Here, indicate whether each material, system or method listed is relevant to your study. If you are not sure if a list item applies to your research, read the appropriate section before selecting a response.

Materials & experimental systems

n/a	Involved in the study	
☒	☐	Antibodies
☒	☐	Eukaryotic cell lines
☒	☐	Palaeontology and archaeology
☒	☐	Animals and other organisms
☒	☐	Clinical data
☒	☐	Dual use research of concern
☐	☒	Plants

Methods

n/a	Involved in the study	
☒	☐	ChIP-seq
☒	☐	Flow cytometry
☒	☐	MRI-based neuroimaging
